# Accurate variant effect estimation in FACS-based deep mutational scanning data with Lilace

**DOI:** 10.1186/s13059-026-03934-1

**Published:** 2026-01-27

**Authors:** Jerome Freudenberg, Jingyou Rao, Matthew K. Howard, Christian Macdonald, Noah F. Greenwald, Willow Coyote-Maestas, Harold Pimentel

**Affiliations:** 1 Bioinformatics Interdepartmental Program, UCLA, Los Angeles, CA USA; 2 Department of Computer Science, UCLA, Los Angeles, CA USA; 3 Department of Bioengineering and Therapeutic Sciences, UCSF, San Francisco, CA USA; 4 Tetrad Graduate Program, UCSF, San Francisco, CA USA; 5 Quantitative Biosciences Institute, UCSF, San Francisco, CA USA; 6 Chan Zuckerberg Biohub, San Francisco, San Francisco, CA USA; 7 Department of Computational Medicine, UCLA, Los Angeles, CA USA; 8 Department of Human Genetics, David Geffen School of Medicine, UCLA, Los Angeles, CA USA

## Abstract

Deep mutational scanning (DMS) coupled with fluorescence-activated cell sorting (FACS) provides a high-throughput method to link genetic variants with quantitative molecular phenotypes. Analysis of these experiments is challenging due to measurement variance and the multidimensional FACS readout. However, no statistical method has yet been developed to address these challenges. Here we present Lilace, a Bayesian statistical model to estimate variant effects with uncertainty quantification from FACS-based DMS experiments. We validate Lilace’s performance and robustness using simulated data and apply it to OCT1 and Kir2.1 DMS datasets, demonstrating an improved false discovery rate while largely maintaining sensitivity.

**Supplementary Information:**

The online version contains supplementary material available at 10.1186/s13059-026-03934-1.

## Background

**Fig. 1:**
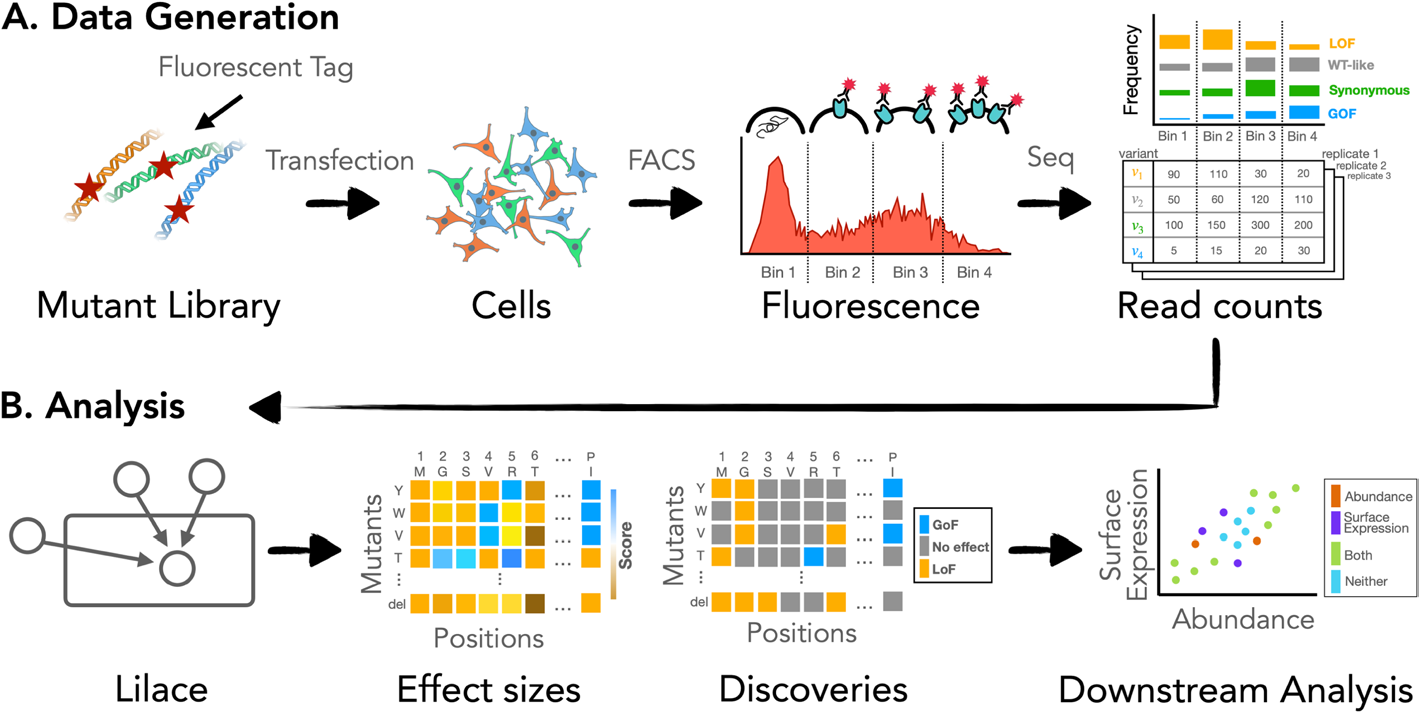
**A** Overview of workflow from experimental process to Lilace input. **B** Uncertainty quantification enables analysis based on statistical significance in addition to effect size

Deep mutational scanning (DMS) experiments help elucidate sequence-function relationships by systematically interrogating the full spectrum of amino acid substitution effects on the molecular properties and function of a protein [1–4]. They provide insight into the mechanisms of protein function and variation [5, 6], as well as aid clinical interpretation of genetic variants [7–9]. When coupled with fluorescence-activated cell sorting (FACS), DMS can scalably measure a quantitative phenotype across variants [1, 10], such as protein abundance [10–15], cell surface expression [12, 16–18], and activity [11, 17, 18]. In such FACS-based DMS experiments, a pooled library of variants is sorted into bins based on fluorescence intensity; then, each bin is sequenced (Fig. 1A). The resulting read counts act as a proxy for the proportion of variants in each bin, representing the tagged phenotype distribution of each variant. To distinguish between variants with approximately wild type-like behavior and those with clear impacts on the phenotype, each variant is compared to a negative control group, such as synonymous variants [13, 14, 19]. To facilitate this comparison, variants are scored on a quantitative scale based on the difference between their phenotype distribution and that of the negative control phenotype.

A highly reproducible and accurate set of scores is essential for ranking high-effect variants to pinpoint functionally relevant protein regions. Additionally, estimating score uncertainty from replicates enables reliability assessment of individual variant scores for potential downstream and clinical interpretation [8, 20]. However, scoring variants based on their FACS readout presents unique statistical challenges, including low sample sizes (two to three replicates is common), experimental noise, and low resolution of the fluorescence distribution due to limited FACS bins (with a standard of four bins). A common scoring approach is to compute a count-weighted mean of integer-labeled bins for each variant [10, 11, 15], then apply a significance cut-off based on the standard deviation of the synonymous variant score distribution [13, 14, 17]. However, determining significance based on a variant’s mean score ignores variability between replicates, does not incorporate read coverage, and typically does not involve an explicit hypothesis test (i.e. *p*-value or analogous test-statistic). The lack of an explicit test results in ad hoc choice of the significance threshold and precludes control of the false discovery rate (FDR), which is necessary to produce reliable results. Another previously used scoring approach applies statistical models designed for growth-based DMS experiments to incorporate measurement uncertainty [14, 17, 20–22]. However, growth-based modeling assumptions, such as a consistent slope across a variant’s bin counts, are misspecified for the FACS readout. In practice, the uncertainty estimates from these models are not used for FACS data [14, 17, 22]. Finally, generalized scoring tools that learn a genotype-phenotype map exist [23–25]; however, these are focused on phenotype prediction, rather than individual variant hypothesis testing.

Here we present Lilace, a Bayesian hierarchical model for FACS-based DMS data (Fig. 1B) that inherently solves the problem of choosing a discovery threshold while providing reproducible and accurate results. The model estimates variant effects relative to a negative control baseline and quantifies effect uncertainties using replicate variance and a negative control-based bias correction. Lilace builds on the efficacy of Rosace [26], an analogous model for growth-based DMS data, and waterbear [27], a model for FACS-based pooled CRISPR screens. Similarly to Rosace, we incorporate a positional effect and a count-variance relationship to improve power in small sample sizes. Similarly to waterbear, we model a latent unimodal shift in the fluorescence distribution to filter experimental noise. We additionally introduce a data normalization procedure to account for disproportionate PCR amplification bias when the FACS gates are not set on equal proportions of the overall fluorescence distribution.

To validate Lilace’s capabilities, we simulated a wide range of experimental designs to quantify and compare discovery set accuracy with previous approaches, which, to our knowledge, we are also the first to benchmark. We simulated from a more complex generative model of the data, inspired by previous FACS simulation designs [27, 28], using simulation parameters estimated from existing datasets. We found that Lilace provided a lower FDR across a variety of simulations, while mostly maintaining sensitivity outside of high experimental variance scenarios. In these high-variance simulations, Lilace continued to provide the lowest FDR and, nevertheless, still retained some sensitivity. We further investigated the performance and generalizability of Lilace by computing empirical performance metrics on real datasets from different experimental procedures, with broad agreement with our simulations. We also examined how Lilace’s uncertainty calculation changed the discovery sets in previous screens of human organic cation transporter OCT1 [14] and inward rectifier potassium channel Kir2.1 [17], corresponding to better agreement with prior biological expectations.

## Results

### Hierarchical Bayesian model for FACS-based DMS data

**Fig. 2:**
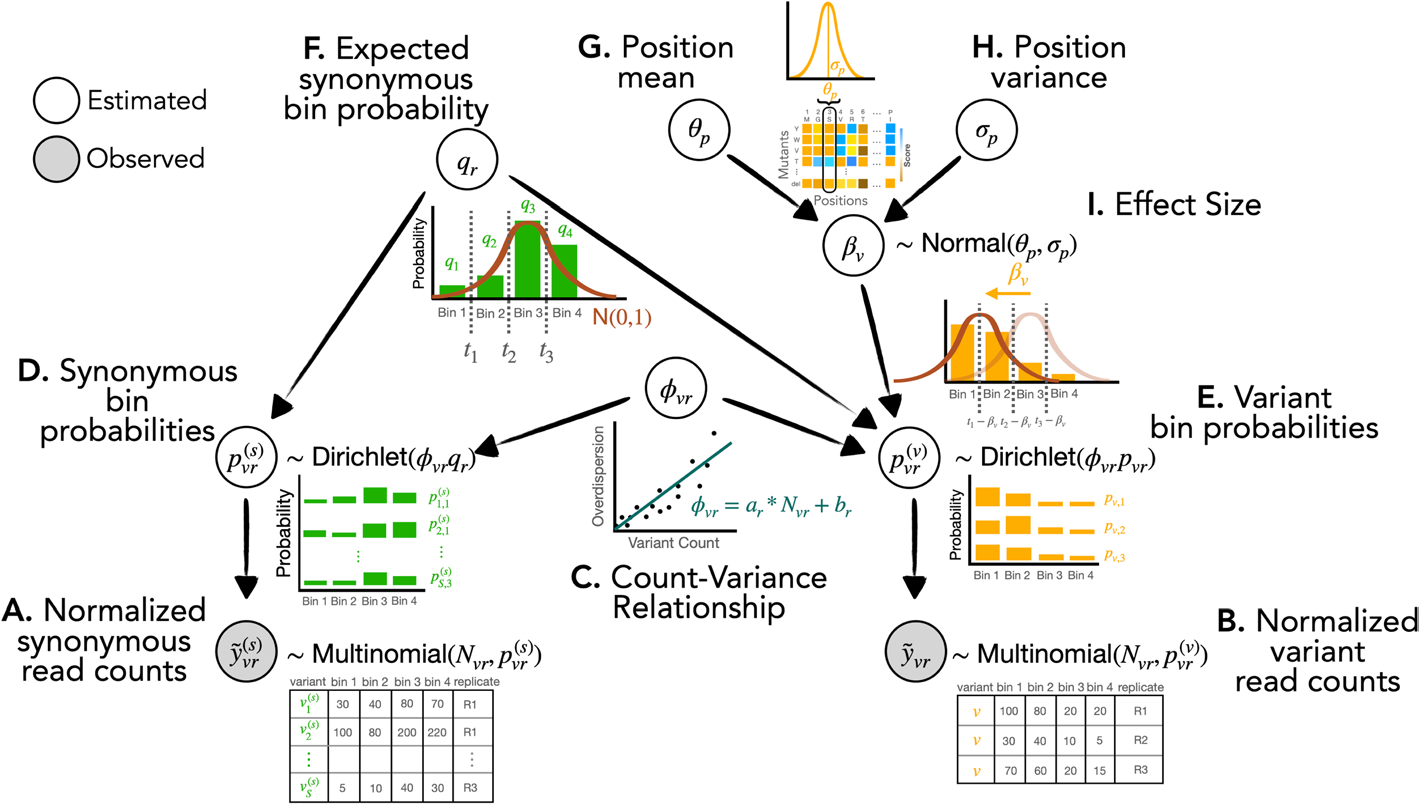
Conceptual visualization of generative model hierarchy (Additional file 1: Fig. S3). **A**, **B** Observed read counts are normalized to cell sorting populations, if available. **C** Overdispersion in read counts is modeled with a count-overdispersion trend function. **D**, **E** Each variant’s read counts are modeled with a latent probability of observing a count in each bin. $p^{(s)}$ and $p^{(v)}$ refer to the sampled synonymous variant and scored variant probabilities, respectively. **F** The expected probability of the negative control group (synonymous variants) within a replicate serves as a baseline. **G**, **H** Variant effect sizes are informed by their position’s score distribution. **I** The effect size is modeled as a shift in the latent bin probabilities between a variant and the negative control baseline

To address the difficulties with statistical inference in FACS-based DMS data, we constructed Lilace to capture the structure of the data in the structure of the model (Fig. 2). Lilace builds on three core ideas: a variant’s effect is manifested as a shift in a unimodal fluorescence distribution, a variant’s count is a noisy observation of the underlying fluorescence distribution, and a variant tends to have similar effects as others at the same residue position. Lilace is fitted using Hamiltonian Monte Carlo (HMC), but for faster inference and larger datasets, we also developed a variational approximation (Methods) [29].

Fundamentally, Lilace places a constraint on the nature of possible changes between individual variant fluorescence distributions, modeling effect sizes as a unimodal shift from the negative control distribution (Fig. 2I, Additional file 1: Fig. S1A). While we use synonymous variants as our negative controls, this group could also, in principle, be composed of a different category of variants, such as a true wildtype. More specifically, Lilace models the bin count proportions for a variant *v* using a latent replicate-specific bin probability $p_{vr}$ (Fig. 2E). The effect size for variant *v* is then modeled as the difference between $p_{vr}$ and the replicate-specific expected bin probability of synonymous variants $q_r$ (Fig. 2F, I). The bin probabilities $p_{vr}$ and $q_r$ are mapped onto corresponding quantile cut-offs in a standard normal and the effect size is fitted as the distance between the cut-offs (Methods). This latent standard normal mapping constrains the possible estimated bin proportions of a variant’s fluorescence distribution to match a unimodal shift from the synonymous fluorescence distribution. Count proportion plots of DMS datasets show that such an effect size trajectory is a reasonable assumption (Additional file 1: Fig. S1B).

Since the observed counts are a noisy representation of the underlying fluorescence distribution, Lilace additionally incorporates an overdispersion parameter $\phi$ to capture this variance, modeled with a count-variance trend function (Fig. 2C). Such count-variance functions are commonly modeled in biological count data [26, 30]–for FACS-based DMS data, we find a linear model to be sufficient (Additional file 1: Fig. S2). Lilace also incorporates the residual variance in synonymous variant scores into the effect size uncertainty as a negative control-based bias correction [31] (Methods). We expect synonymous variants to display wild type-like behavior [32], so we can use the variance in their scores to help distinguish variants between wild type-like and those with sufficient evidence of an effect.

Lastly, Lilace incorporates positional information to regularize effect estimates using a position-specific mean and variance (Fig. 2G, H). This regularization allows the model to leverage the position mean as an informative prior for variants with less data, while the variance parameter prevents stronger or well-supported effects from being incorrectly pulled toward that mean. Sharing information within a position via this Bayesian hierarchy was shown by Rao et al. (2024) to improve sensitivity in DMS datasets [26]. While we expect modeling position-level effects to improve estimation for most experiments, as observed in Rosace [26], we also provide the option to run Lilace without this hierarchy for positionally unbiased estimates. Although this decreases overall performance, it would prevent potential underdetection of weak signals opposite to their position mean. Additionally, to avoid excessive shrinkage in the synonymous mutation effect sizes and preserve their use as negative controls, Lilace incorporates a fixed effect for each synonymous mutation with a single shared variance term. This approach keeps the relative ranking of their scores consistent with their count observations among all variants (Additional file 1: Fig. S4).

### Lilace has improved FDR with comparable sensitivity

**Fig. 3:**
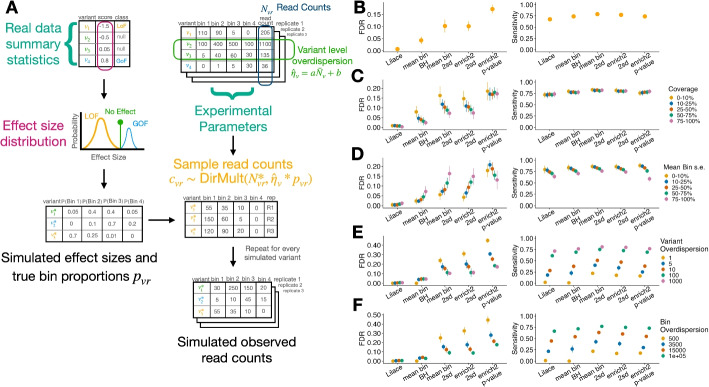
**A** Conceptual visualization of generative model used for simulations. The effect distribution and experimental noise parameters for the simulation were estimated from an input dataset. **B**-**F** FDR and sensitivity metrics from OCT1 abundance-seeded simulations. Panel (**B**) shows the overall performance on the data-estimated default setting, which is then partitioned by (**C**) read coverage percentile and (**D**) mean bin standard error percentile. Performance robustness was measured by varying (**E**) variant overdispersion and (**F**) bin overdispersion. Standard error bars were computed over ten iterations initialized with different random seeds. Remaining parameter figures and Kir2.1 surface expression-seeded simulation results are included in Additional file 1: Figs. S5 and S6. Remaining method comparisons are included in Additional file 1: Fig. S7

To examine our model’s performance against a known ground truth, we developed simulations informed by marginal parameter distributions from real data (Methods). We set default simulation parameters based on parameter estimates from an input dataset, then varied each parameter individually to examine how different sources of dataset heterogeneity affected model performance (Fig. 3A). As input datasets for our simulation, we used an abundance screen on OCT1, performed with a protocol based on VAMP-seq [10], and a surface expression screen on Kir2.1, performed with a fluorescent antibody tagging protocol [12]. Using our simulated ground truth, we then benchmarked the FDR and sensitivity of Lilace with previously applied approaches, including the most widely used growth-based DMS tool Enrich2 [20], a custom maximum likelihood-based scoring approach based on previous Sort-seq estimators [33], and a weighted bin average [10, 11] that uses the synonymous variant score distribution to determine a discovery threshold. The latter two act as a general proxy for methods that attempt to either estimate the actual variant fluorescence or non-parametrically score variants using a mean of integer-labeled bins, since implementations can vary in practice. For both of these approaches, we benchmarked a typical two synonymous standard deviation discovery threshold (95% confidence interval), the Benjamini-Hochberg (BH) corrected version of this threshold, a replicate-specific *p*-value combination approach, and a t-test (Methods).

While most methods generally had strong correlations with the true simulated effect sizes, Lilace outperformed the other methods in higher replicate or bin-level variance configurations (Additional file 1: Figs. S8, S9, S10). The mean bin approach also struggled to capture the full range of effect sizes, saturating earlier than other methods (Additional file 1: Fig. S11). Additionally, the ML approach displayed instability in its effect estimation in several settings.

In the data-estimated default setting, we found that Lilace achieved the lowest FDR, with around a 90% reduction from the next best performing method (Fig. 3B, Additional file 1: Figs. S5, S6, S7). Lilace and the BH-corrected mean bin approach were the only two methods to provide adequate FDR control at a 0.05 cutoff. While the BH-corrected mean bin approach controlled the overall FDR to 0.05, we note the BH correction does not guarantee even distribution of false discoveries. Since the mean bin approach is unable to take into account coverage level and replicate variance, we observed higher FDR concentration among variants with low counts (Fig. 3C) or high variance (Fig. 3D). This pattern was especially impactful among variants close to the mean bin threshold (Additional file 1: Fig. S12), leading to substantially increased FDR among smaller effect discoveries.

Lilace continued to provide better FDR across simulation configurations resulting from both biological and experimental variability (Fig. 3E, F, Additional file 1: Figs. S5, S6). This improvement in FDR was especially pronounced in configurations with high variance between individual variant observations (Fig. 3E), bin sequencing variance (Fig. 3F), or a low proportion of variants with an effect (Additional file 1: Figs. S5, S6). We also found that variational Lilace performed similarly to Lilace, outside of these high variance configurations (Additional file 1: Fig. S13).

Lilace generally retained power to detect effects, but prioritized FDR control, resulting in marginally lower sensitivity. In the default simulation, Lilace had slightly lower sensitivity than the highest sensitivity approaches (Fig. 3B, Additional file 1: Figs. S5, S6). We suspected this was driven by the large proportion of small effects in the default simulation (Methods). Filtering to the top 50% of effects mitigated this decrease, supporting this hypothesis and indicating that Lilace remained well-powered to detect more impactful variants (Additional file 1: Fig. S14). We further explored this FDR-sensitivity trade-off across approaches by examining discovery threshold-agnostic performance. For example, setting the mean bin discovery threshold to greater synonymous standard deviations would also reduce sensitivity in favor of lower FDR. We found that while Lilace performed the best in this metric, most approaches had a similar overall trade-off (Additional file 1: Fig. S15). However, a main strength of Lilace lies in adaptively adjusting discovery classifications at a 95% certainty cut-off to keep FDR consistent in noisy data, whereas fixed discovery threshold approaches do not have this flexibility. As a result, Lilace is more robust to sources of experimental variance.

We also benchmarked performance across FACS-related gating parameters, including number of bins and gate offset (Additional file 1: Figs. S5, S6). In our simulation defaults, we set FACS gates to contain an equal proportion of the overall fluorescence distribution; however, gating can be done differently, especially when examining multiple conditions [22]. For example, gating thresholds could be determined in an inactive protein state, shifting the fluorescence distribution of the active protein toward the rightmost bins. We found that severely shifting the gates from the equal proportion default hurt performance for all approaches (Additional file 1: Figs. S5, S6). This behavior is expected, since differentiating effects is harder if they are manifested within a smaller range of bins. Other approaches had a resulting increase in FDR and a decrease in sensitivity, while Lilace again retained a consistently low FDR.

**Fig. 4:**
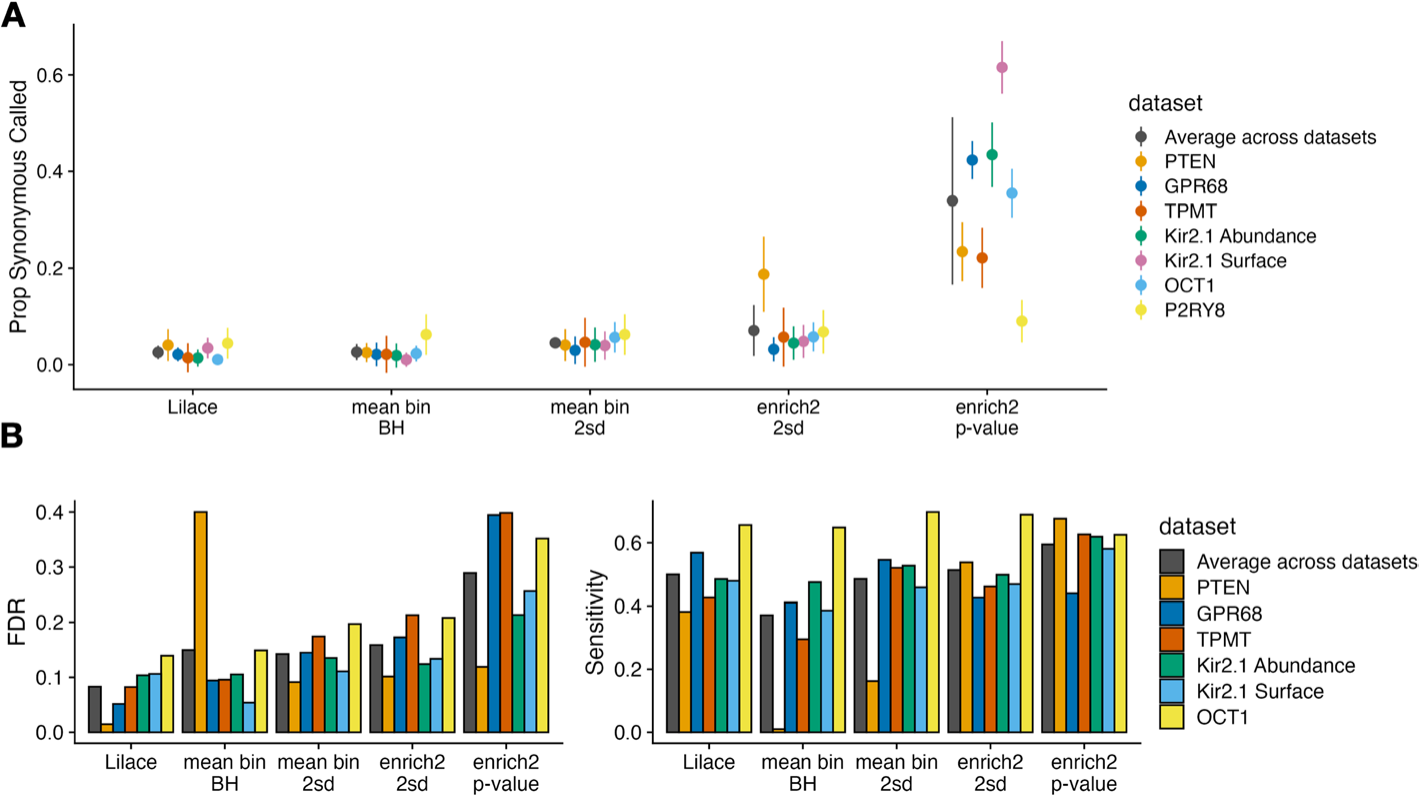
Empirical FDR and Sensitivity results for each discovery calling approach as determined by (**A**) masking 20% of the synonymous mutations to estimate FDR across 10 iterations and (**B**) using AlphaMissense pathogenicity labels across the first 6 datasets to determine relative FDR (benign label) and Sensitivity (pathogenic label). ROC curves for each dataset are shown in Additional file 1: Fig. S16. The BH correction vastly reduced the PTEN mean bin’s discovery set leading to the poor calibration (Additional file 1: Fig. S17). The P2RY8 dataset tracked poorly with AlphaMissense for all discovery calling approaches and is included separately in Additional file 1: Fig. S18

In addition to simulations, we examined a more direct empirical proxy for FDR in real datasets by randomly masking 20% of synonymous mutations, treating them as any other variants, and checking how many were called incorrectly. We applied this analysis to a range of previously published FACS-based screens to benchmark generalizability, including OCT1, Kir2.1, GPR68, PTEN, TPMT, and P2RY8 [10, 12, 14, 18, 22]. We found that Lilace and the BH-corrected mean bin again provided the best FDR using this metric (Fig. 4A, Additional file 1: Fig. S7). Since there is no analogous positive control set for high-effect variants, we instead used AlphaMissense pathogenicity predictions as an imperfect proxy for tracking relative method performance [34]. We found that Lilace had a lower FDR derived from benign predictions and comparable sensitivity derived from pathogenic predictions, affirming our simulation conclusions (Fig. 4B, Additional file 1: Figs. S7, S19). Lilace effect sizes also had the highest or equivalent to the highest correlation with the AlphaMissense pathogenicity scores for every dataset tested (Additional file 1: Table S1), suggesting that Lilace can reduce noise in variant scores. We also empirically assessed sensitivity using nonsense variants and ClinVar pathogenic-labeled variants, where available (Additional file 1: Fig. S20) [35], with similar results.

### Lilace trims the discovery set in an OCT1 abundance screen

**Fig. 5:**
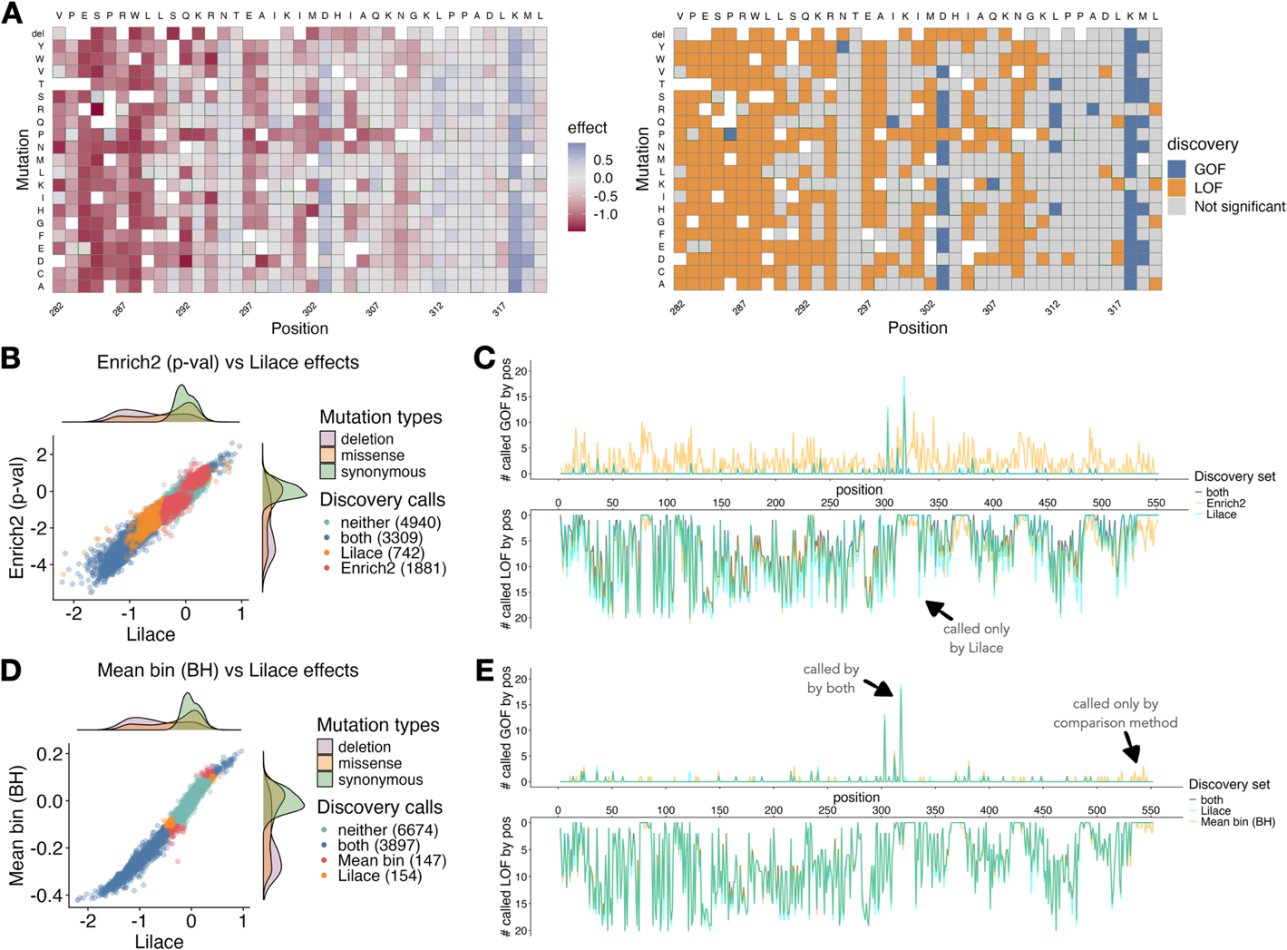
**A** Sample Lilace effect size and discovery heatmaps for OCT1 positions 282–320 at a significance threshold of lfsr < 0.05. In the discovery heatmap, orange colors indicate a significant LOF effect and blue colors indicate a significant GOF effect. **B** Score and discovery difference between Lilace and Enrich2 (*p*-value based), colored by whether a variant is discovered by both approaches, only Lilace, only Enrich2, or neither. **C** Position-wise discovery differences with Enrich2’s discovery set, comparing the number of significant GOF (upper) and LOF (lower) hits at each position. Orange indicates additional discoveries by the original approach, cyan indicates Lilace, and gray indicates both, with a resulting teal for overlapping discovery calls. **D**-**E** The same analysis as in B and C repeated for the BH-corrected weighted mean bin scoring approach

To qualitatively examine discovery differences, we compared Lilace’s results with those of previous approaches in the datasets used to seed our simulations. Lilace’s OCT1 abundance discovery set generally agreed with analysis from Yee et al. that used a two standard deviation cutoff of the Enrich2 scores, identifying the N-terminal transmembrane domains (TMs) as enriched for discoveries and several C-terminal TMs as depleted (Additional file 1: Fig. S21) [14]. The discovery heatmap separates the regions enriched and depleted for effects (Fig. 5A, Additional file 1: Fig. S21), exemplifying how filtering by variant effect uncertainty can clarify biologically relevant patterns.

Compared to the BH-corrected mean bin approach, which had the next best FDR in simulations, Lilace did not call around 4% (147/4044) of its discoveries (Fig. 5D). Compared to the standard mean bin approach, Lilace did not call 13% (586/4625) of its discoveries (Additional file 1: Fig. S22). These variants had sufficiently large variance in their effect sizes such that there was insufficient evidence for Lilace to call them as discoveries, reducing the overall discovery set. A stronger pattern in discovery differences emerged when compared with using the Enrich2 *p*-values, which is the only commonly applied approach that incorporates replicate variance and calls discoveries based on statistical significance. We expect Enrich2 to be misspecified for FACS data, as estimating a slope across bins does not take into account the shape of the underlying fluorescence distribution. This misspecification was reflected in the *p*-values, which only had a 0.68 correlation with the Lilace local false sign rates (lfsrs). In this case, we found that Lilace did not call around 36% (1881/5190) of the Enrich2-based discoveries (Fig. 5B). Additionally, we found Enrich2 had 24% (2623/10872) overall discovery call disagreement with Lilace, whereas the BH-corrected and standard mean bin approaches had 3% (301/10872) and 6% (601/10872) disagreement, respectively.

The reduction in called discoveries was especially noticeable in putative gain-of-function (GOF) variants, which are expected to be milder [36] and less frequent [1] than loss-of-function (LOF) effects. Lilace identified 95 total GOF effects, compared to 144 with the BH-corrected mean bin approach (Fig. 5E), 352 using the standard mean bin approach (Additional file 1: Fig. S22), and 1200 using Enrich2 (Fig. 5C), with notable reduction in the terminal domains (Fig. 5C, E). Additionally, Lilace-identified GOF effects were largely concentrated at positions 303 and 318, of which the former was validated in follow-up experiments [14].

Lilace also identified an additional 154 and 742 variants compared to the BH-corrected mean bin approach (Fig. 5D) and Enrich2 (Fig. 5B), respectively. These were mainly LOF and scattered in positions with at least one other LOF substitution, demonstrating the effect of position-level shrinkage (Fig. 5C, E). We also found an additional GOF variant, I392F, which was not identified by the mean bin-based or Enrich2-based approaches and escaped negative position-level shrinkage (−0.54 position effect).

### Lilace trims discovery sets in different Kir2.1 phenotypes

To further examine Lilace’s generalizability and potential for discovery, we applied Lilace to the Kir2.1 dataset [12, 37]. Whereas the OCT1 experiment was based on the VAMP-seq platform with fluorescent protein fusion to measure abundance, the Kir2.1 screen instead used an antibody fluorescent tagging protocol to measure surface expression and abundance [12]. Since abundance is a precursor to surface expression, we expect there to be reasonable agreement between the two phenotypes, except for variants that affect only surface expression. The integration of discovery sets from both of these phenotypes illustrates the effect of Lilace’s discovery differences on downstream analyses such as phenotype comparison.

**Fig. 6:**
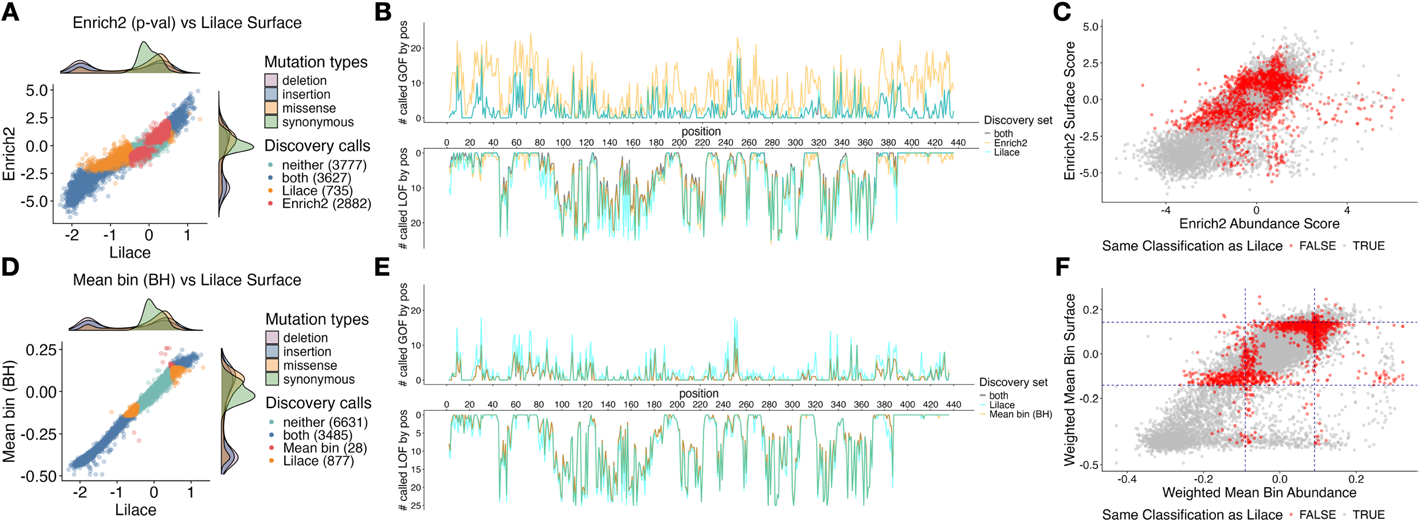
Lilace score and discovery classification differences with (**A**-**C**) Enrich2 *p*-value and (**D**-**F**) BH-corrected weighted mean bin for Kir2.1. **A**, **B** and **D**, **E** are Kir2.1 surface expression discovery comparison analogues to the previous OCT1 abundance plots. **C**, **F** compare the effects of both phenotypes on their original respective effect sizes, colored by disagreement with the Lilace discovery set. In (**F**), the BH-corrected cutoff on the original scores is plotted as a dashed line, showcasing the added flexibility of incorporating variant measurement uncertainty into the decision boundary

The Lilace discovery set highlighted the expected functional regions of Kir2.1, with much of the LOF signal concentrated in the pore domain and golgi export-related residues (Additional file 1: Figs. S23, S24, S25) [17]. In both phenotypes, we found an overall reduced discovery set compared to the Enrich2 and standard mean bin approaches (Fig. 6A, Additional file 1: Figs. S26A, S27), consistent with our previous results showing Lilace’s prioritization of FDR control leads to a smaller discovery set. A substantial portion of the removed GOF discovery calls again came from the C-terminal domain (Fig. 6B, Additional file 1: Figs. S26B, S27), suggesting that the original calls could have been due to technical variance rather than biological signals. We observed greater measurement variance in this region, supporting this conclusion (Additional file 1: Fig. S28). While Lilace was more conservative in calling weaker-evidenced variants, it also called 392 additional variants across both phenotypes compared to the standard mean bin approach and 1080 additional variants compared to Enrich2. The most concentrated and consistent increase in discoveries occurred as additional LOF calls in the surface expression phenotype at positions 382–387 (Fig. 6B, E, Additional file 1: Fig. S27B), which houses a previously identified diacidic ER-export motif [12, 17, 38].

Compared with the BH-corrected mean bin scoring approach, Lilace had around 5% (588/11021) and 8% (905/11021) disagreement for the abundance and surface expression phenotype discovery calls, respectively (Fig. 6D, Additional file 1: Fig. S26). Interestingly, Lilace identified an additional 25% (877/3513) more variants than the BH-corrected mean bin approach in the surface expression phenotype (Fig. 6D). Based on the differences between these two methods in simulations, we examined the coverage (Additional file 1: Fig. S29) and mean bin standard error (Additional file 1: Fig. S30) distributions of their discovery sets. We found that the mean bin standard errors for this phenotype tended to be smaller than the OCT1 abundance data, with the extra Lilace calls concentrated in the lower standard error region, suggesting that the additional discoveries could be due to greater confidence in variants with smaller replicate variance.

The impact of a changed discovery set is compounded when model output is combined for downstream analysis, such as comparing phenotypes for mechanistic inference. When using our significance cut-off to classify variants as affecting only protein abundance, only surface expression, both, or neither, we found a 47% (5232/11021) overall difference with the Enrich2 *p*-value-based discovery set (Fig. 6C) and a 13% overall difference with both the BH-corrected (1400/11021) and standard mean bin-based (1419/11021) sets (Fig. 6F). Furthermore, compared to the mean bin-based approaches, Lilace identified a lesser proportion affecting only abundance and a greater proportion impacting only surface expression or both phenotypes (Additional file 1: Table S2). These proportions align more with the phenotype hierarchy, since we expect variants that affect abundance to have a downstream effect on surface expression, but not the other way around.

## Discussion

Previously, no general statistical method had been specifically designed to score FACS-based phenotypes with uncertainty quantification [21], despite fluorescence being one of the most common readouts for DMS experiments. Furthermore, the performance of previous ad hoc scoring approaches had not been quantified, leaving their validity and generalizability across datasets unverified. As a result, variant scores from these experiments often come without thorough quantification of their reliability and reproducibility, such as via standard errors and measures of significance like *p*-values. The lack of reliability and reproducibility can lead to inefficiency validating or interpreting variants with unknown score uncertainty. We fill this method gap with Lilace, a Bayesian hierarchical model that adapts its discovery classifications based on measurement uncertainty, improving on the reliability of previously applied ad hoc approaches.

Lilace leverages replicate variance and FACS-specific distributional assumptions for inference of variant effect size uncertainty, while using synonymous variant scores as an internal negative control set to mitigate false discoveries. Our results demonstrate that Lilace offers improved FDR control and robustness, based on both simulations and empirical benchmarks. For situations where Lilace’s runtime is prohibitive, such as large multi-protein or multi-phenotype datasets, variational Lilace offers a substantially faster alternative (Additional file 1: Fig. S31), at the cost of increased FDR (Additional file 1: Fig. S19). The BH-corrected mean bin approach can also provide a reasonable discovery set; however, it does not account for differences in read coverage and between-replicate variance, leading to lower efficacy in some datasets. On the other hand, Enrich2’s uncertainty quantification does not perform well in any scenario, which is consistent with the misalignment of growth-based modeling assumptions with FACS data.

By prioritizing FDR control, Lilace often outputs a reduced discovery set in real data, especially in putative gain-of-function effects, likely due to fewer false positives and slightly lower sensitivity to small effects. While this mild decrease in sensitivity appears consistent, we expect that the large reduction in FDR will facilitate clearer interpretation of effects. Additionally, when the proportion of small effects is low, Lilace has no loss of sensitivity (Additional file 1: Figs. S5, S6, S14). Model extensions could help mitigate the sensitivity decrease with a less conservative inclusion of synonymous effect variance, such as an auxiliary model to learn the bias distribution from the negative controls [31]. Improving synonymous variance incorporation could also improve the power gain from position-level shrinkage, since the shrinkage will apply to a greater proportion of the effect variance.

Like with Rosace, the position-level shrinkage boosts discovery power, without introducing substantial bias that prevents the identification of variant effects opposite to their position mean [26]. Analogously to Rosace, this position grouping is only applicable to single or fixed mutation datasets, without straightforward application to random mutagenesis experiments with multiple mutations. While it is possible to run Lilace without the position-level grouping, properly extending Lilace to these datasets is an important area of future work. Although uncommon, a dataset may also not have a distribution of synonymous variants to use as a negative control. While it would still be possible to score against a given baseline proportion, such as a true wildtype or the overall fluorescence distribution, Lilace’s negative control-based bias correction is not possible and performance would likely echo that of unrecalibrated Lilace (Additional file 1: Fig. S7).

Recent work in DMS modeling has additionally incorporated priors on the effects of amino acid substitutions, which could be similarly incorporated into Lilace [39, 40]. Other sources of prior information such as structure or protein language model embeddings could also be incorporated. Finally, while we use cross-phenotype analysis to demonstrate the impact of improved discovery sets, a joint analysis framework is needed to fully model multi-phenotype relationships.

Although there is more work to be done, we hope that Lilace will enable researchers to obtain reliable, reproducible, and interpretable scores for FACS-based DMS experiments. This is especially critical as experiments scale to greater numbers of measurements across conditions, phenotypes, and proteins, making ad hoc analysis impractical. As we demonstrate in the multi-phenotype analysis, even seemingly small FDR differences can compound to change downstream interpretations of variant effects. We expect that more robust DMS analysis tools can aid functional dissection of genetic variant effects at every level, including protein mechanisms [1, 2], human phenotypes [5, 6], and clinical interpretation [7–9, 41, 42].

## Conclusions

Lilace fulfills the need for a statistical model to estimate variant scores in FACS-based DMS experiments. Through improved FDR control and effect size estimation over standard approaches, Lilace enables more accurate evaluation of these scores, enhancing interpretability and reliability. As DMS experiments scale to larger sets of measurements and begin to be leveraged clinically, reliable effect size interpretation becomes increasingly important. We anticipate that Lilace will serve as an essential tool to tackle this challenge.

## Methods

### Data processing

Processed count datasets used for our analyses were received from their respective original publications [10, 12, 14, 18, 22]. For the OCT1, Kir2.1, GPR68, and P2RY8 datasets, reads had been trimmed, error-corrected, and mapped using BBTools [43], then aggregated using GATK’s AnalyzeSaturationMutagenesis tool [44]. For the PTEN and TPMT datasets, the reads had been processed using ea-utils [45] and were aggregated over barcode-linked variants using Enrich2 [20]. Reads were aggregated into a single count for each amino acid change, including synonymous variants, within each bin and replicate. As a pre-processing step, we filtered out variants with less than 15 overall counts across replicates. We also added a pseudocount to help with model fitting.

In the scenario of unequal cell proportions in each bin, PCR amplification can lead to disproportionately large counts for bins with lower proportions. To account for this bias, we used the cell proportions from the FACS sort reports to normalize each bin’s counts to match its cell proportion (with the exception of PTEN and TPMT as sort reports were not available) [46]. Given a variant *v* from replicate *r* with *K* bins, we rescaled the bin counts in bin *k*, $y_{vrk}$, based on the replicate and bin-specific cell proportions $\pi _{rk}$, the total read counts in that bin $R_{rk}$, and the total read counts across bins $R_r$.

1 $$\begin{aligned} \tilde{y}_{vrk} = \left\lceil {y_{vrk} \left(\frac{\pi _{rk} R_r}{R_{rk}}\right)}\right\rceil \end{aligned}$$

This results in a length *K* vector of rescaled bin counts for each variant and replicate: $\tilde{y}_{vr}$. While this normalization is necessary to avoid sequencing-induced bias in the effect sizes, it can also amplify noise for very low count variants, leading to larger standard errors (Additional file 1: Fig. S32). This could further decrease power to detect effects in these variants. In the absence of cell sorting information for this normalization, Lilace’s use of a replicate-specific scoring baseline can still help account for this bias (Additional file 1: Fig. S33).

### Model details

Lilace models the bin counts $\tilde{y}_{vr}$ from variant *v* and replicate *r* as,

2 $$\begin{aligned} \tilde{y}_{vr} \sim \text {DirichletMultinomial}(N_{vr}, \phi _{vr} p_{vr}) \end{aligned}$$

where $N_{vr}$ is the sort proportion-normalized variant read count, $\phi _{vr}$ is the overdispersion term, and $p_{vr}$ is the variant’s latent bin probability across *K* bins. We model $\phi _{vr}$ using a linear trend function of the variant counts, estimated within each replicate,

3 $$\begin{aligned} \phi _{vr} = a_r + b_r N_{vr} \end{aligned}$$

To estimate an effect size for each variant from *K* gating bins, we model the replicate-specific baseline fluorescence bin probability of the negative controls (synonymous variants) $q_r$ and estimate the distance of each $p_{vr}$ from $q_r$ as a shift in the corresponding probability quantiles of a standard normal,

4 $$\begin{aligned} \tilde{y}_{vr}^{(s)}\sim & \text {DirichletMultinomial}(N_{vr}, \phi _{vr} q_r) \end{aligned}$$

5 $$\begin{aligned} t_{r,k}= & \boldsymbol{\Phi }^{-1}\left( \sum \limits _{i=1}^k q_{ri}\right) \end{aligned}$$

6 $$\begin{aligned} p_{vrk}= & \left\{ \begin{array}{ll} \Phi (t_{r,1} - \beta _v) & k=1 \\ \Phi (t_{r,k} - \beta _v) - \Phi (t_{r,k-1} - \beta _v) & k \in [2,K-1] \\ 1 - \Phi (t_{r,K-1} - \beta _v) & k = K \end{array}\right. \end{aligned}$$

where $y^{(s)}$ is a subset of *y* containing only the synonymous control variants, $t_{r,k}$ is the standard normal quantile cutoff corresponding to bin *k*’s baseline probability in replicate *r* for $k \in [1, K-1]$, and $\Phi (\cdot )$ is the standard normal CDF. The effect size $\beta _v$ is the shift in these cutoffs to match variant *v*’s bin probabilities. Similar latent normal quantile approaches have previously been successfully applied to FACS-based CRISPR screens [27, 47, 48]. We jointly estimate $q_r$ and $\beta _v$ during estimation to capture variance in the baseline cutoffs. We find that modeling a replicate-specific baseline instead of a global *q* can account for bias due to replicate-specific shifts in the counts, such as due to unequal sorting or PCR amplification (Additional file 1: Fig. S33).

We incorporate position level hierarchy by sampling effects from a position-specific distribution:

7 $$\begin{aligned} \beta _v \sim N\left(\theta _p, \sigma _p^2\right) \end{aligned}$$

where $\theta _p$ and $\sigma _p^2$ represent the mean and variance of the effects at position *p*. This parameterization results in position-specific shrinkage, meaning effects incorporate a prior bias towards their position’s mean effect to help estimation in low sample size variants. While effect shrinkage is helpful for estimating variant true effects, we aim to avoid excessive shrinkage in synonymous variants to preserve their interpretation as a negative control group. To this end, Lilace encodes a fixed effect for each synonymous mutation, so that each has their own mean, while still sharing a single variance term.

8 $$\begin{aligned} \beta _s \sim N\left(\theta _s, \sigma _0^2\right) \end{aligned}$$

where *s* indexes the set of synonymous mutations. We then use the individual posteriors of each synonymous mutation for negative control-based bias correction on our effect sizes [31]. We incorporate the variance between synonymous mutations into our decision boundary to separate variants into those that act as wild type-like and those with a substantial impact. Without this effect recalibration, we find that the model does not have adequate FDR control (Additional file 1: Fig. S7). Let *D* be the number of posterior samples and *S* be the number of synonymous variants. For each posterior sample $d \in 1,\dots ,D$ and variant *v*, we select a random synonymous variant $s_{vd}$ to recalibrate that posterior sample’s score.

9 $$\begin{aligned} \beta _{vd}^{*} = \beta _{vd} - \beta _{s_{vd} d} \end{aligned}$$

Our new score is $\hat{\beta }_v = \frac{1}{D}\sum _{d=1}^D \beta ^{*}_{vd}$ with variance $\hat{\sigma }^2_v = \text {Var}(\beta ^{*}_v)$. To classify an effect as a discovery we use the local false sign rate (lfsr) of the corrected posterior $\beta ^{*}_v$ [49], with a default discovery threshold of 0.05.

The position and synonymous mean parameters $\theta _p$ and $\theta _s$ are given *N*(0, 1) priors. The variance parameters $\sigma _p$ and $\sigma _0$ are given $\text {InvGamma}(1,1)$ priors. The baseline threshold probabilities $q_r$ are given a $\text {Unif}(0,1)$ prior. All other parameters are given a flat uniform prior. We fit Lilace with MCMC sampling using the default NUTS algorithm for HMC [50] in Stan [51]. On most proteins, Lilace runs within a couple of hours (Additional file 1: Fig. S31).

We implemented the variational version of Lilace using stochastic variational inference (SVI) in Pyro [52]. To adapt the model to Pyro, we made two changes to our priors: we replaced the flat improper uniform priors on $a_r$ and $b_r$ with $\text {HalfNormal}(0.1, 10)$ priors and the $\text {Unif}(0,1)$ prior on $q_r$ with a $\text {Dirichlet}(1,...,1)$ prior. We relied on Pyro’s AutoGuide class to define the variational families, testing both a normal (AutoNormal) and multivariate normal (AutoMultivariateNormal) guide parameterization. For the results in this paper, we trained the model for 5000 steps, using the Adam optimizer [53] with a learning rate of 0.001, and 3 random restarts.

### Other method details

We ran Enrich2 with each bin encoded as a timepoint and using the sum of synonymous variant counts in each bin as the wildtype reference. We used either the provided *p*-value with a Benjamini-Hochberg FDR correction [54] or standard deviations from the synonymous score as the discovery threshold.

For the weighted mean bin approach, we computed the score for a variant as:

10 $$\begin{aligned} w_{vr}= & \frac{\sum _k\frac{k}{K}(y_{vrk})}{\sum _k (y_{vrk})} \end{aligned}$$

11 $$\begin{aligned} \mu _v^{(\text {mean bin)}}= & \frac{1}{R} \sum \limits _r w_{vr} \end{aligned}$$

12 $$\begin{aligned} \mu _{\text {syn}}^{(\text {mean bin)}}= & \frac{1}{S}\sum \limits _{v \in \text {syn}} \mu _v^{(\text {mean bin)}} \end{aligned}$$

13 $$\begin{aligned} \beta _{v}^{(\text {mean bin)}}= & \mu _v^{(\text {mean bin)}} - \mu _{\text {syn}}^{(\text {mean bin)}} \end{aligned}$$

For the maximum-likelihood approach, we estimated the underlying fluorescence distribution using the raw FACS gating thresholds by minimizing the negative log-likelihood of a log-normal [33].

14 $$\begin{aligned} NLL_v = -\left( \sum \limits _{r} \sum \limits _{k} y_{vrk} \text {log}(F_{\mu _v, \sigma _v}(U_k) - F_{\mu _v, \sigma _v}(L_k))\right) \end{aligned}$$

where *F* is the cumulative distribution function for a log-normal, $U_K$ and $L_k$ represent the upper and lower FACS gating thresholds for bin *k*, and $\mu _v$ and $\sigma _v$ represent the mean and variance parameters of the log-normal.

We then computed a score as the difference in means between the average mean fluorescence of a variant and that of synonymous variants:

15 $$\begin{aligned} \mu _{\text {syn}}^{(\text {ML)}}= & \frac{1}{S}\sum \limits _{v \in \text {syn}} \mu _v \end{aligned}$$

16 $$\begin{aligned} \beta _v^{(\text {ML)}}= & \mu _v - \mu _{\text {syn}}^{(\text {ML)}} \end{aligned}$$

For both the weighted mean bin and maximum-likelihood based approaches, we implemented numerous mechanisms for estimating uncertainty. In our primary approach, we fitted a normal distribution to the synonymous variant scores to calculate *p*-values, with the typical two standard deviation cutoff corresponding to a *p*-value cutoff of 0.05, which were then adjusted with the BH procedure. However, while this effect thresholding approach is most commonly used in the literature, it does not attempt to account for replicate variance. To include benchmarks that account for this, we also performed a Welch’s t-test for each variant, as well as combined within-replicate tests using *p*-value combination approaches. For the t-test approach, we estimated:

17 $$\begin{aligned} \nu= & \frac{\left(\frac{s_v^2}{N_v} + \frac{s_s^2}{N_s}\right)^2}{\frac{s_v^4}{N_v^2 (N_v-1)}+\frac{s_s^4}{N_s^2 (N_s-1)}} \end{aligned}$$

18 $$\begin{aligned} t= & \frac{\bar{X}_v - \bar{X}_s}{\sqrt{s_{\bar{X}_v}^2 + s_{\bar{X}_s}^2 }} \end{aligned}$$

where $\nu$ is the degrees of freedom, $\bar{X}_v$ and $s_v$ are the estimated mean and corrected sample standard deviation across replicates for variant *v*, $\bar{X}_s$ and $s_s$ are the synonymous mean and corrected sample standard deviation across replicates, $N_v$ is the number of replicates for variant *v*, and $N_s$ is the number of synonymous variant observations across replicates.

For the ML *p*-value combination approach, we first fitted a log-normal to the synonymous variants within each replicate by minimizing:

19 $$\begin{aligned} NLL_{r}^{\text {(syn)}} = -\left( \sum \limits _s\sum \limits _{k} y_{srk} \text {log}(F_{\mu _{r}^{\text {(syn)}}, \sigma _{r}^{\text {(syn)}}}(U_k) - F_{\mu _{r}^{\text {(syn)}}, \sigma _{r}^{\text {(syn)}}}(L_k))\right) \end{aligned}$$

where *s* indexes the set of synonymous variants in replicate *r*. Then, we fitted log-normals for each variant *v* within each replicate *r* by minimizing:

20 $$\begin{aligned} NLL_{vr} = -\left( \sum \limits _k y_{vrk} \text {log}(F_{\mu _{vr}, \sigma _{vr}}(U_k) - F_{\mu _{vr}, \sigma _{vr}}(L_k))\right) \end{aligned}$$

We then calculated a *p*-value for each variant within a replicate using a likelihood ratio test between the synonymous-fitted parameters and the variant-specific parameters:

21 $$\begin{aligned} \lambda _{vr} = -2\left(l\left(\mu _{r}^{\text {(syn)}}, \sigma _{r}^{\text {(syn)}} | y_{vr}\right)-l\left(\mu _{vr}, \sigma _{vr} | y_{vr}\right)\right) \end{aligned}$$

where the log-likelihood function *l* is defined as in Eq. 20 and $\lambda _{vr}$ is chi-square distributed with 2 degrees of freedom. We then combined these *p*-values across replicates into a single variant *p*-value using Simes or Fisher’s *p*-value combination methods, before applying a BH correction. For the mean bin *p*-value combination approach, we computed individual *p*-values for $w_{vr}$ using a normal distribution fitted to that replicate’s synonymous mean bin estimates. We then combined these *p*-values using the same approach as in the ML case.

### Simulation methods

To compare and benchmark Lilace with simulated data, we designed a separate generative model of the experimental process that parameterizes various sources of natural and technical variance. Since this model was used only for simulation purposes, we did not need to ensure identifiability and implemented a more parameterized model. We marginally estimated simulation model parameters from real data to provide more realistic defaults, while varying each one across a range of values to examine the relative performance of our model with other approaches. We determined discoveries based on a 95% certainty threshold for all approaches (0.05 *p*-value threshold for frequentist methods).

Our simulation procedure was as follows:

1. Input protein and experimental summary statistics
2. Simulate ground truth effect sizes
3. Simulate variant cell fluorescence and bin read counts
4. Add experimental variance in observed counts

We first picked an input protein dataset and set default simulation constants based on this protein including *V* variants per position, *R* replicates, *L* total read counts, and *K* FACS bins. We also set the default number of cells per variant, *m*, to 200. For simulation efficiency, we fixed the number of positions *P* to 100.

To estimate the effect size distribution from data, we applied the LogNormal MLE approach, since these effect sizes are on the same scale as the underlying fluorescence distribution’s parameters. However, since the individual variant effect sizes were unreliable due to low sample size, we only used the summary statistics of the collective effect distribution to parameterize our simulation. To enable the investigation of different effect size architectures via varying the proportions of small effects, we estimated separate distributions for small and large effects. We first fit a bivariate Gaussian mixture model to the maximum-likelihood derived effect sizes to estimate a distribution of small effects and a distribution of large effects, labeling the small effect mode as the one containing more synonymous variants. From the fitted mixture model, we derived the proportion of small effects $p_{\text {small}}$ and fit a Gaussian to each mode to derive the parameters of both effect groups, $\mu _{\text {small}}$, $\sigma _{\text {small}}$, $\mu _{\text {large}}$, $\sigma _{\text {large}}$. Note that with this procedure many of the small effects are derived from variants that are unlikely to have a significant effect in the real data, but incorporating simulated effects of such a low magnitude helps compare scoring approaches in benchmarking. Next, we simulated from these fitted groups with $\beta _{\text {small}} \sim N(\mu _{\text {small}}, \sigma _{\text {small}})$ and $\beta _{\text {large}} \sim N(\mu _{\text {large}}, \sigma _{\text {large}})$. We truncated the effects to be within 2 standard deviations of their group mean to remove outliers and create a greater distinction between the groups.

We then used these effect sizes to generate ground truth effect sizes in a position-specific manner. We parameterized the position effect as the variance in effects at a position $\sigma _p$, which we estimated from the MLE-based effect sizes. At each position *p*, we decided the effect pattern based on the real data’s effect pattern *W* determined using the weighted mean bin approach. Every synonymous variant or variant that is not identified as an effect in the real data was assigned a zero effect size. We sampled the rest of the effects by first uniformly sampling a position mean $\theta _p$ from our effect size distribution, with a probability $p_{\text {small}}$ of sampling from $\beta _{\text {small}}$ and a probability $1 - p_{\text {small}}$ of sampling from $\beta _{\text {large}}$. Then, we sampled the effects at that position as $\beta _p \sim N(\theta _p, \sigma _p)$. Under large $\sigma _p$, the proportion of small and large effects at the position could change; however, on average these proportions stayed consistent across the simulated dataset.

22 $$\begin{aligned} W_{pj} = \mathbbm {1}\left\{ \text {real data effect at position}\ p\ \text {and variant}\ j\right\} \end{aligned}$$

23 $$\begin{aligned} \beta _{pj} \sim \left\{ \begin{array}{ll} N(\theta _p, \sigma _p) & (W_{pj} = 1 \text { and non-synonymous}) \\ 0 & (W_{pj} = 0 \text { or synonymous}) \end{array}\right. \end{aligned}$$

To optionally vary the overall proportion of effects, we took *W* and randomly increased or decreased the effects until the target effect proportion was reached.

To simulate each variant’s latent fluorescence distribution, we first used the set of synonymous variants to estimate the fluorescence distribution of wild type-like variants using the Log-Normal MLE approach, treating each synonymous count observation as an independent observation of the same distribution. Let $\mu _0$ and $\tau$ be the wild type-like Log-Normal location and scale parameters. For each simulated variant *v*, we sampled *m* cells from the latent fluorescence observation $F_v$ as:

24 $$\begin{aligned} F_v= & \text {Log-Normal}\left(\mu _0 + \beta _v, \tau ^2\right) \end{aligned}$$

25 $$\begin{aligned} f_v\sim & F_v \end{aligned}$$

When simulating a replicate effect *B*, we added a constant term $b_r$, derived from length *R* equidistant steps in $[-B,B]$, to the log-normal location parameter in replicate *r*. We then set gating thresholds as equally spaced quantiles of the overall fluorescence distribution $\sum _v f_v$ and divided cell counts into sorting bins accordingly. We used those latent sorted cell counts to set the true bin probability for a variant $p_{vr}$.

26 $$\begin{aligned} p_{vrk} = \frac{1}{m} \sum \limits _{i=1} ^m\mathbbm {1} \{L_k< f_{vi} < U_k\} \end{aligned}$$

where $L_k$ and $U_k$ are the lower and upper fluorescence bounds of bin *k*, respectively. When simulating shifted gates, we shift non-endpoint $L_k$ and $U_k$ by a constant gate offset term on a log scale (to match the log normal effect magnitudes).

To generate the observed counts, we incorporated variant-specific technical variance by sampling from a Dirichlet-Multinomial with overdispersion parameter $\eta _v$. Since only a handful of replicates is insufficient to estimate the parameters of a Dirichlet-Multinomial, we used the set of synonymous variants to estimate the relationship between variant read count and $\eta _v$. We first estimated the expected synonymous bin probabilities

27 $$\begin{aligned} q_k = \frac{\sum _r\sum _{v \in \text {syn}} \frac{y_{vrk}^{(s)}}{\sum _{k^*} y_{vr{k^*}}^{(s)}}}{RS} \end{aligned}$$

where *R* is the number of replicates and *S* is the number of synonymous variants.

To get estimates of $\eta _v$, we used Brent’s method with a lower bound of zero and an upper bound of 1000 to minimize the negative log-likelihood:

28 $$\begin{aligned} \hat{\eta }_v = \mathop {\arg \min }\limits _{\eta _v \in (0,1000)} - \sum \limits _{v \in \text {syn}} \sum \limits _r \text {log} f\left(y_{vr}^{(s)} | N_{vr},\eta _v*q\right) \end{aligned}$$

where *f* is the Dirichlet-multinomial likelihood and the bin proportions *q* are fixed to their expected value. We then regressed these estimates of $\eta _v$ on the average replicate read count for that variant $\bar{N}_v$ to get a linear estimate of the count-variance function.

29 $$\begin{aligned} \hat{\eta }_v = a*\bar{N}_v + b \end{aligned}$$

For each replicate, we then randomly sampled a read count $N_{vr}^*$ from the seed dataset, plugged $N_{vr}^*$ into Eq. 29 to estimate $\hat{\eta }_v$, and simulated variant *v*’s observed bin read counts as

30 $$\begin{aligned} c_{vr} \sim \text {DirichletMultinomial}\left(N_{vr}^*, \hat{\eta }_v * p_{vr}\right) \end{aligned}$$

where $c_{vr}$ is variant *v*’s cell count in replicate *r* across *K* bins.

When benchmarking performance across bin overdispersion parameters, e.g. due to PCR amplification bias, we instead set $c_{vr}$ to the simulated sorted cell counts, then sample the reads from bin *k* with an input overdispersion parameter $\psi _k$:

31 $$\begin{aligned} p_{vrk}= & \frac{c_{vrk}}{\sum _{v^*} c_{{v^*}rk}} \end{aligned}$$

32 $$\begin{aligned} c_{{\bullet } {\bullet } k}^{\text {(obs)}}\sim & \text {DirichletMultinomial}\left( \frac{N}{K}, \psi _k * p_{{\bullet }{\bullet }k}\right) \end{aligned}$$

We used the resulting counts to benchmark our model’s performance and make relative comparisons with other approaches. When varying specific parameters, we set that parameter to a specific value and left the others at their data-estimated defaults. Each simulation setting was repeated 10 times to obtain average FDR and sensitivity statistics with standard errors.

### Empirical benchmarking

For our synonymous-based empirical benchmarking (Fig. 4A), we randomly masked 20% of synonymous mutations in each dataset tested, treating them as any other variant at their position. We estimated FDR by computing the fraction of masked synonymous variants called and ran this procedure 10 times with different masks to get standard errors. For the AlphaMissense-based benchmarking (Fig. 4B, Additional file 1: Fig. S16), we retrieved available AlphaMissense pathogenicity predictions and scores for each protein tested [55], then used benign labels as a proxy true negative and pathogenic labels as a proxy true positive to compute FDR and sensitivity on. For the ClinVar-based sensitivity benchmarking of Kir2.1 and PTEN (Additional file 1: Fig. S20), we used missense variants labeled “likely pathogenic” or “pathogenic” as our proxy true positive set.

## Supplementary Information

Additional file 1. Supplementary Figures and Tables. Contains additional simulation and real data analyses referenced in the main text.

## Data Availability

Lilace is available to run as an R package and can be installed from https://github.com/pimentellab/lilace [56], where it is distributed under an MIT License. Lilace VI is implemented in a separate python script and can be found linked at the same location. Archived versions of Lilace and Lilace VI are available on Zenodo [57, 58]. Retrieved count data and code used for simulations and analyses can be found at https://github.com/jermoef/lilace-paper-analysis [59]. Original datasets for OCT1 [14, 60], Kir2.1 [12, 61], GPR68 [22, 62], PTEN [10, 63], TPMT [10, 63], and P2RY8 [18, 64] can be retrieved from their respective publications. ClinVar pathogenicity labels [35] (https://www.ncbi.nlm.nih.gov/clinvar/) and AlphaMissense scores [55] (https://alphamissense.hegelab.org/) were obtained from their respective online resources.
