# Supplementary material for: Accurate variant effect estimation in FACS-based deep mutational scanning data with Lilace
Source: Genome Biol. 2026 Jan 27;27:48. doi: 10.1186/s13059-026-03934-1 (PMC12918140; doi:10.1186/s13059-026-03934-1)
Supplement: Supplementary file 1 — Additional file 1. Supplementary Figures and Tables. Contains additional simulation and real data analyses referenced in the main text. [file 13059_2026_3934_MOESM1_ESM.pdf]

Supplementary Figures and Tables

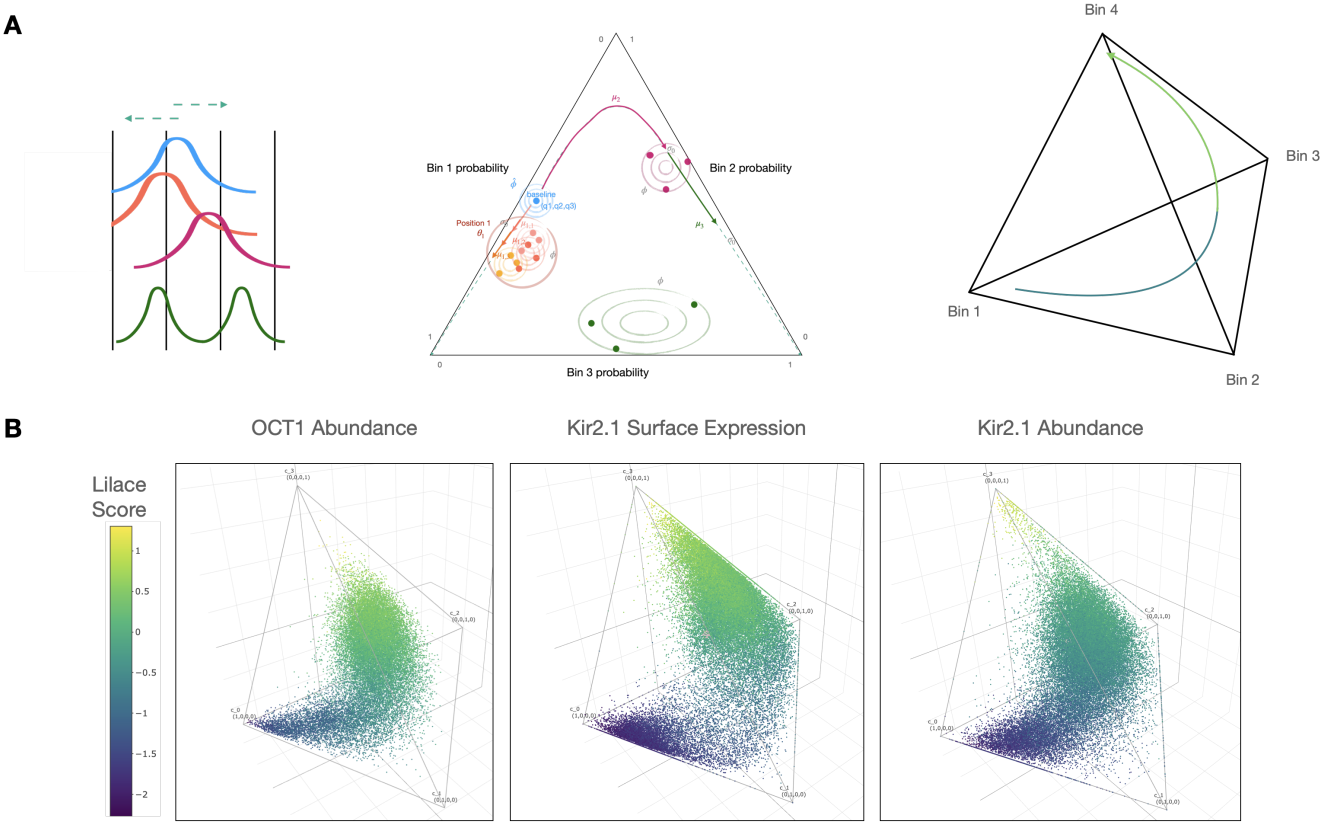

Figure S1: (A) Visual representation of latent effect size trajectory in probability space for $K = 3$ or $K = 4$. (B) Plots of the raw count data proportions in the same space depict a similar trajectory across variants. Each variant observation is a point that is colored by its score, with low scores having a large count proportion in the lowest bin (c_0) and high scores in the highest bin (c_4). A bimodal variant would be opposite of the curve (most counts spread across the first and last bin)–we can see there are very few, if any, such variants, and they are assigned a moderate score.

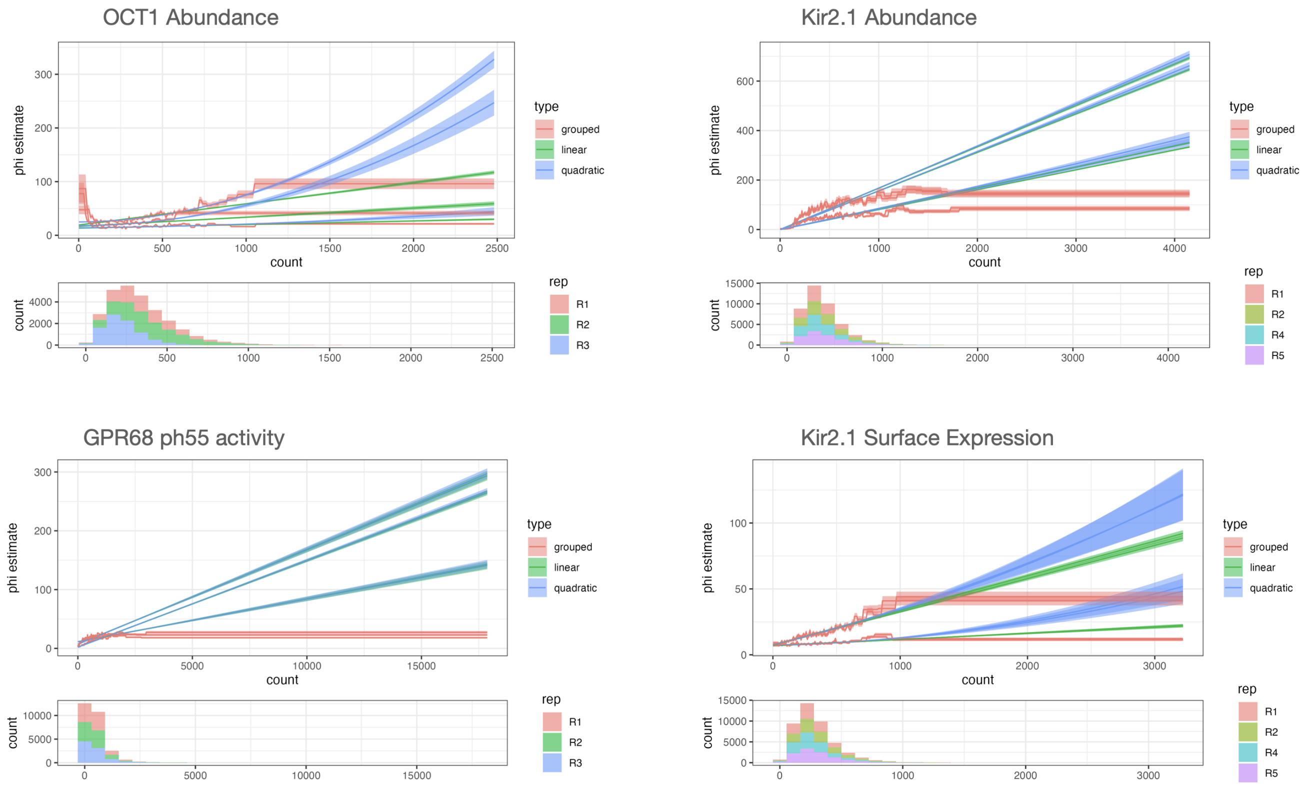

Figure S2: Comparison of count-overdispersion trend function estimates. In the regime of counts where almost all variant observations are located, the linear function approximation is similar to a employing a quadratic approximation or a Rosace-like non-parametric count grouping of size 100 each.

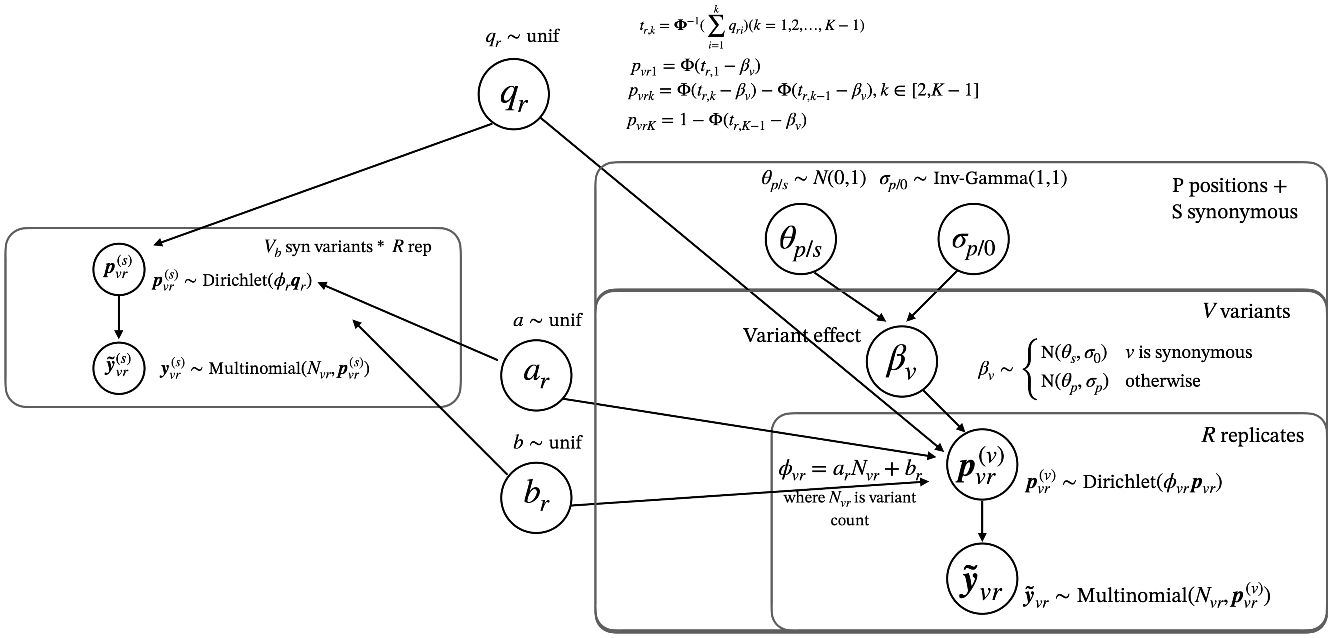

Figure S3: Bayesian plate model for Lilace that matches main Figure 2. The effect size β_v is influenced by its position mean θ_p and variance σ_p^2. Synonymous variants are not grouped with their position and are instead given their own fixed effect θ_s with a single shared variance term σ_0^2. β_v measures the distance in latent probabilities between synonymous variants q and the variant p_v. The baseline bin proportion for each replicate, q_r, is estimated from synonymous variants $y^{(s)}$ and their associated probabilities $p^{(s)}$. Each variant's baseline bin proportion, p_{vr}, is estimated from the variant's counts y_{vr} and their associated probabilities $p^{(v)}$. See Methods for a detailed model description.

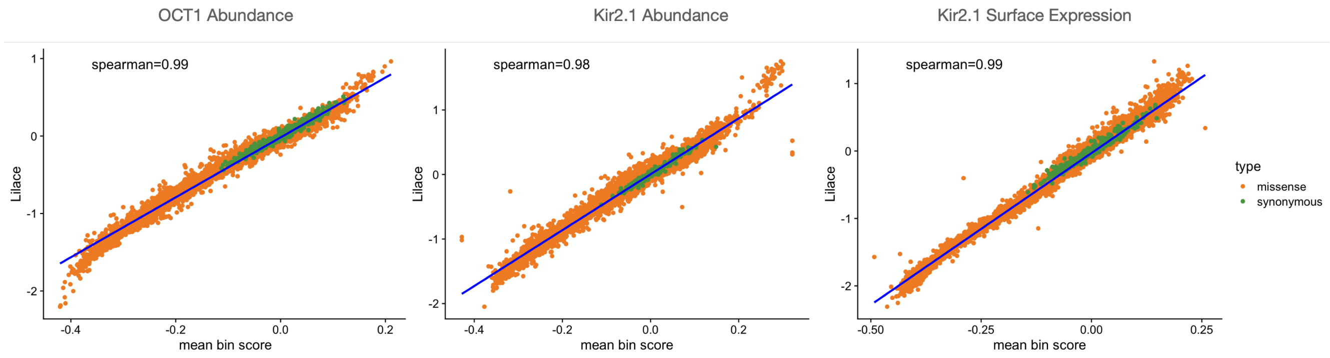

Figure S4: Bin rank consistency between Lilace and the weighted mean bin approach

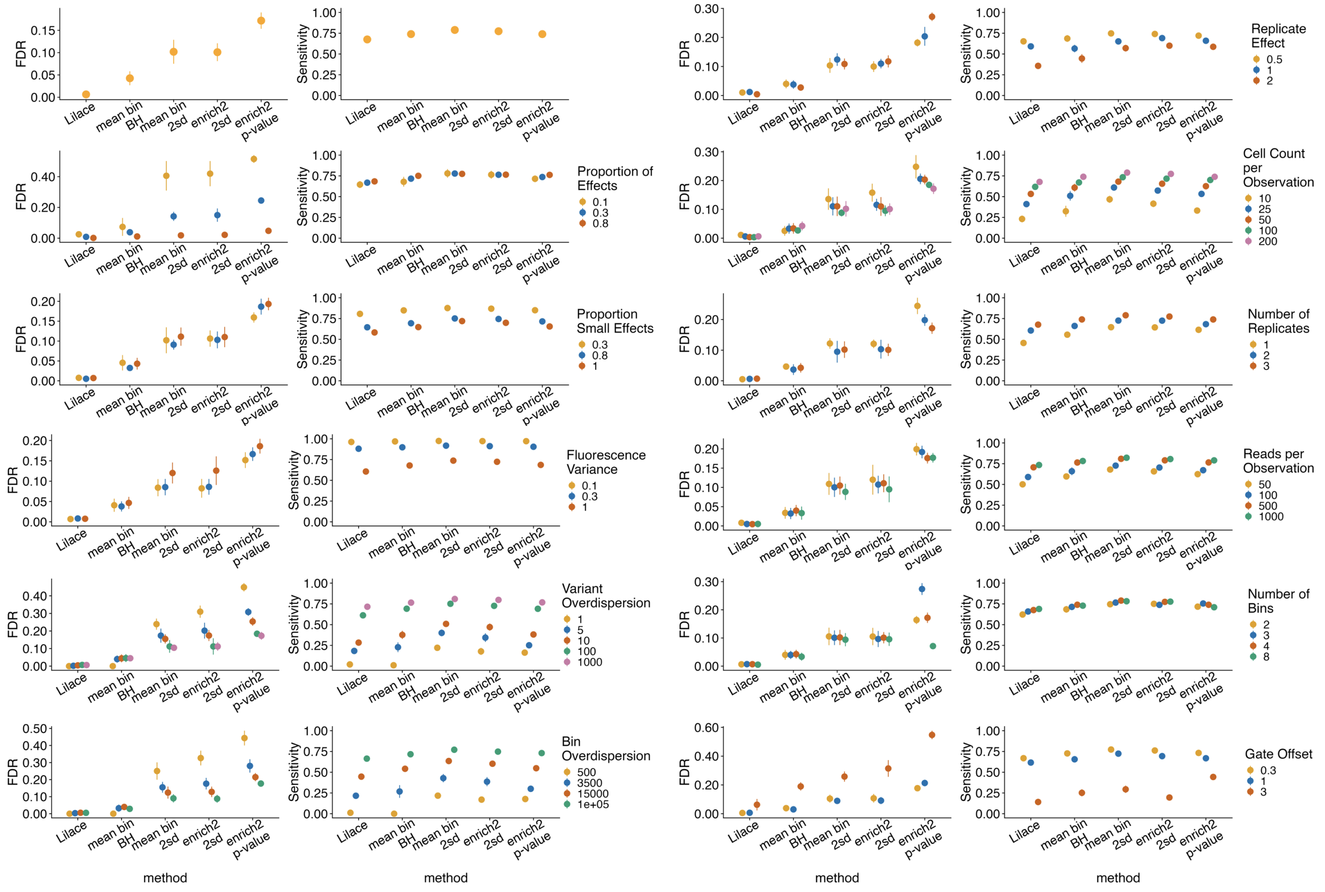

Figure S5: Full OCT1 Abundance Simulations

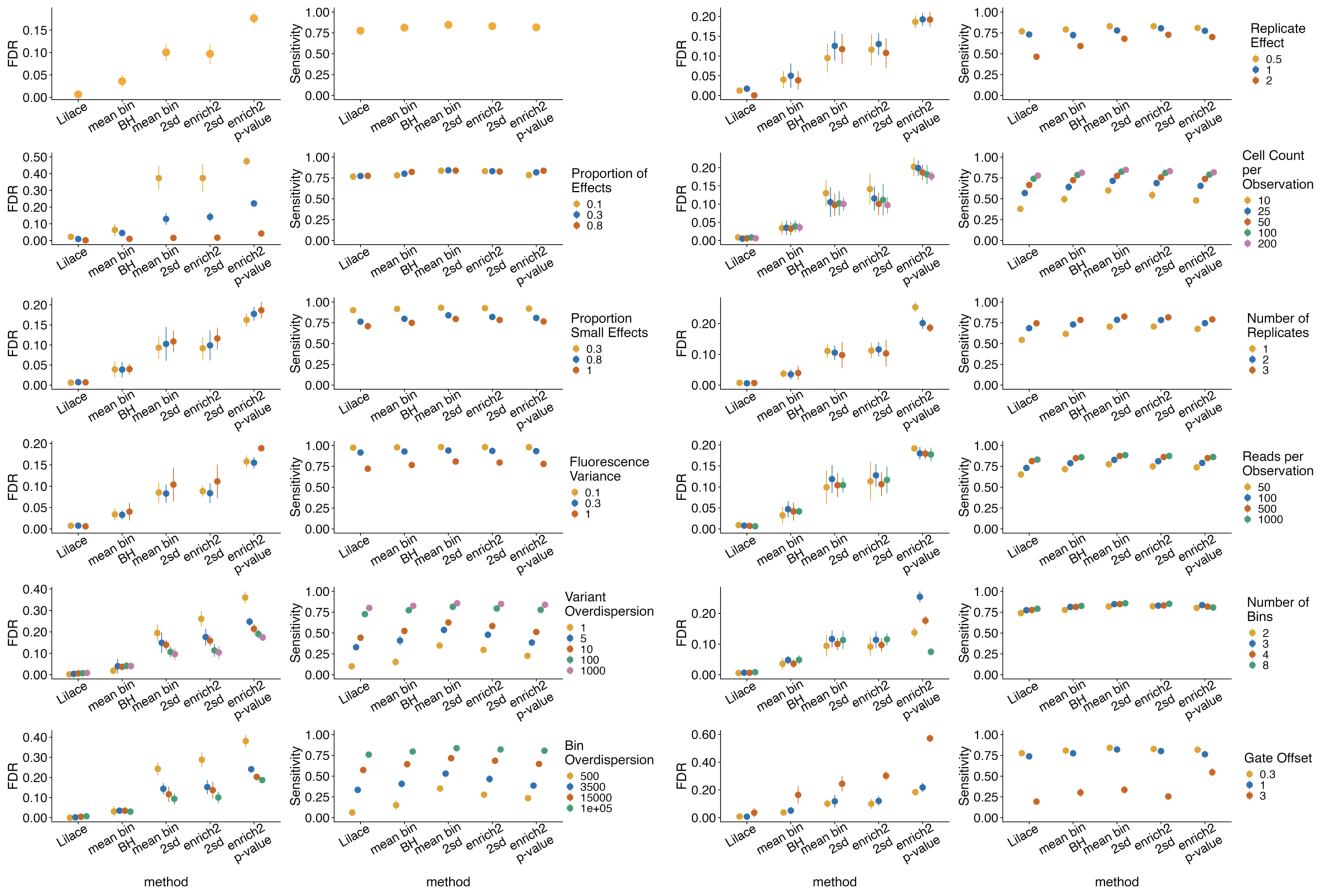

Figure S6: Full Kir2.1 Surface Expression simulations

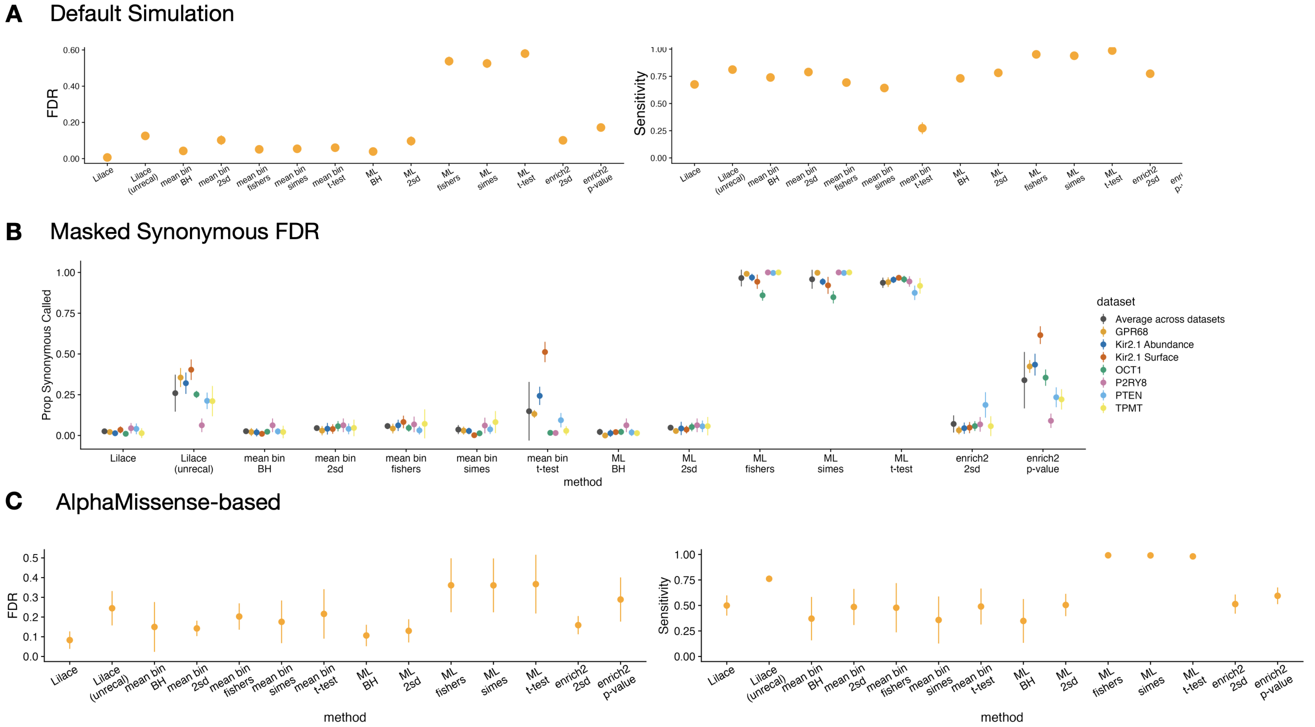

Figure S7: (A) Full method comparison set in the default simulation setting, including unrecalibrated Lilace and approaches utilizing replicate variance based on the mean bin and ML approaches (see Methods). (B) Full method comparison in the masked synonymous mutation analysis. (C) Full method comparison in the AlphaMissense-based analysis (average across datasets).

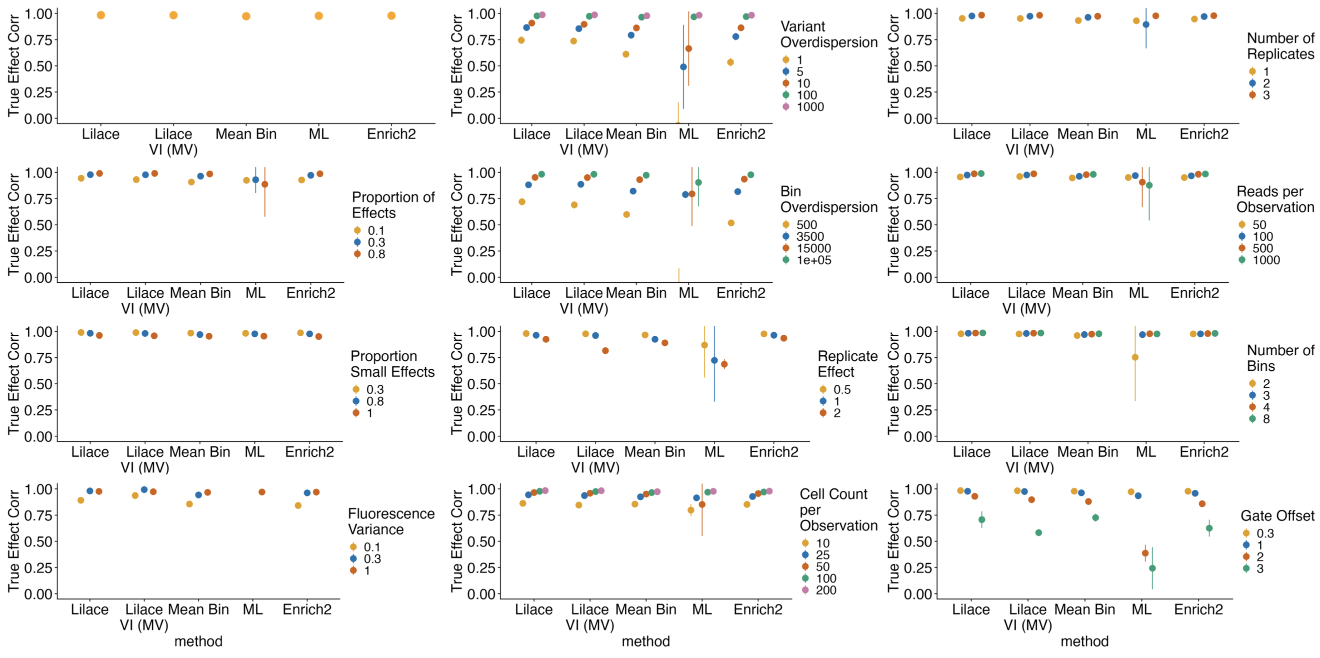

Figure S8: Correlations with ground truth effect sizes across OCT1-based simulation configurations

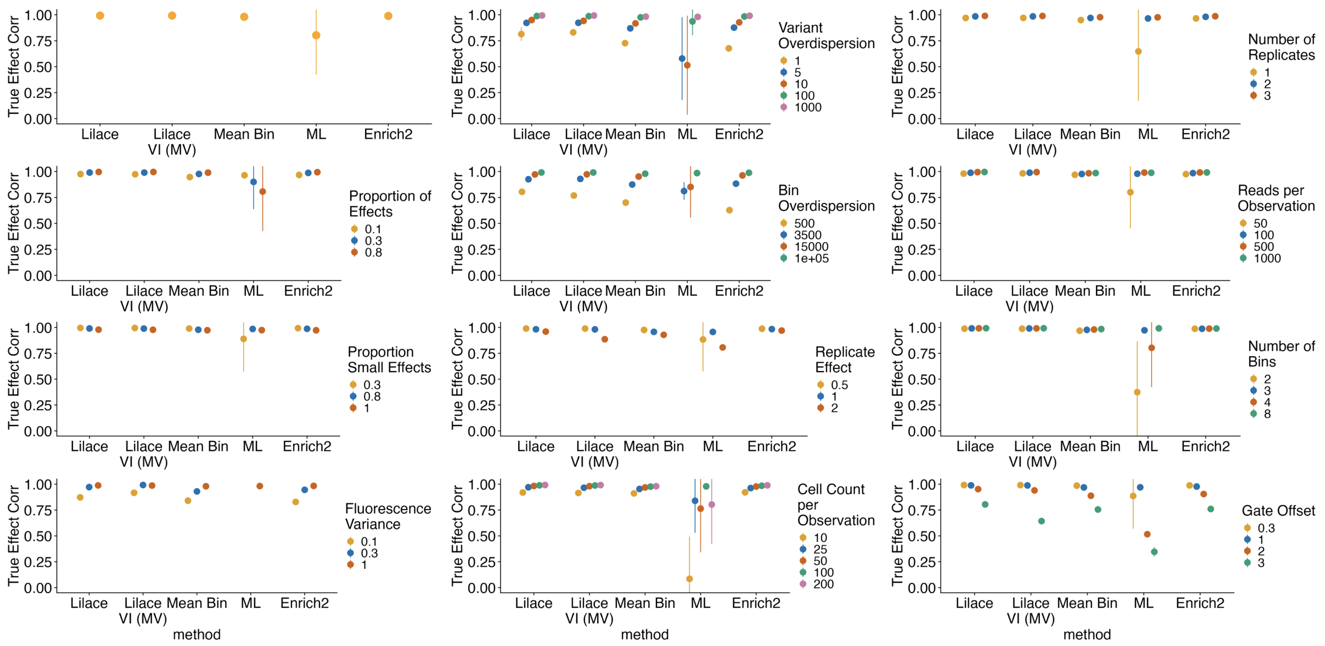

Figure S9: Correlations with ground truth effect sizes across Kir2.1-based simulation configurations

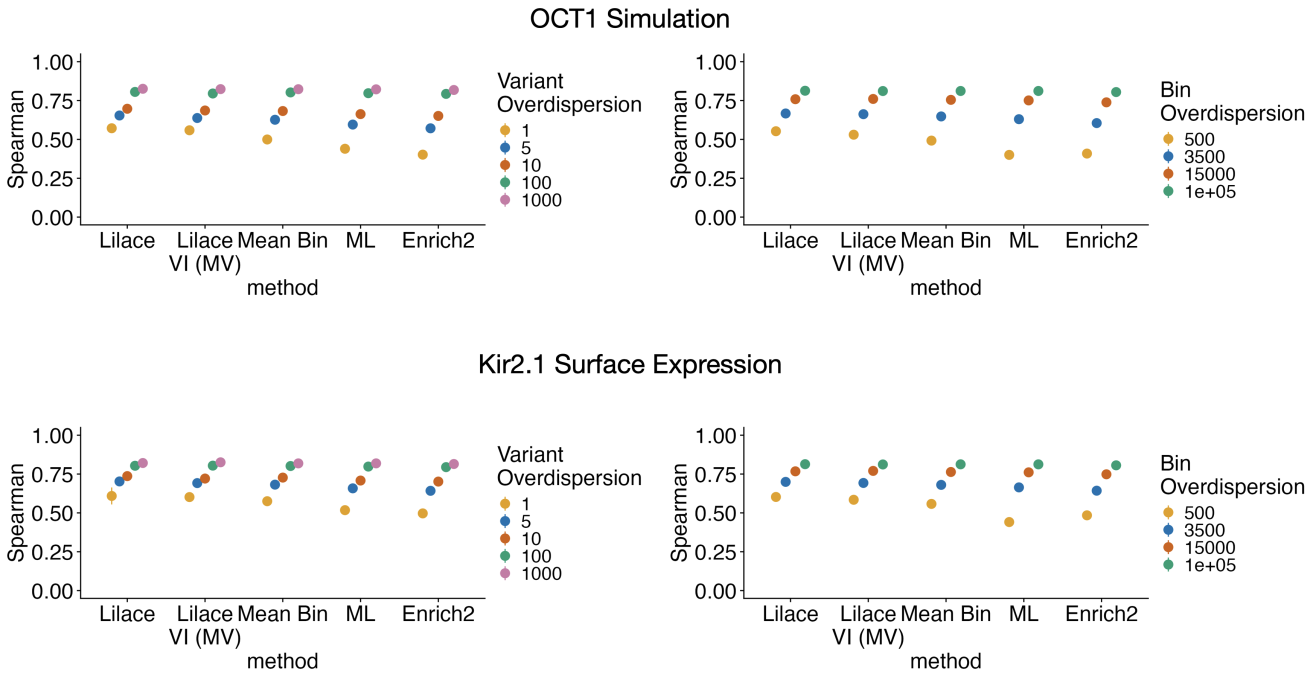

Figure S10: Spearman correlations with ground truth effect sizes for overdispersion configurations from both OCT1-based and Kir2.1-based simulations

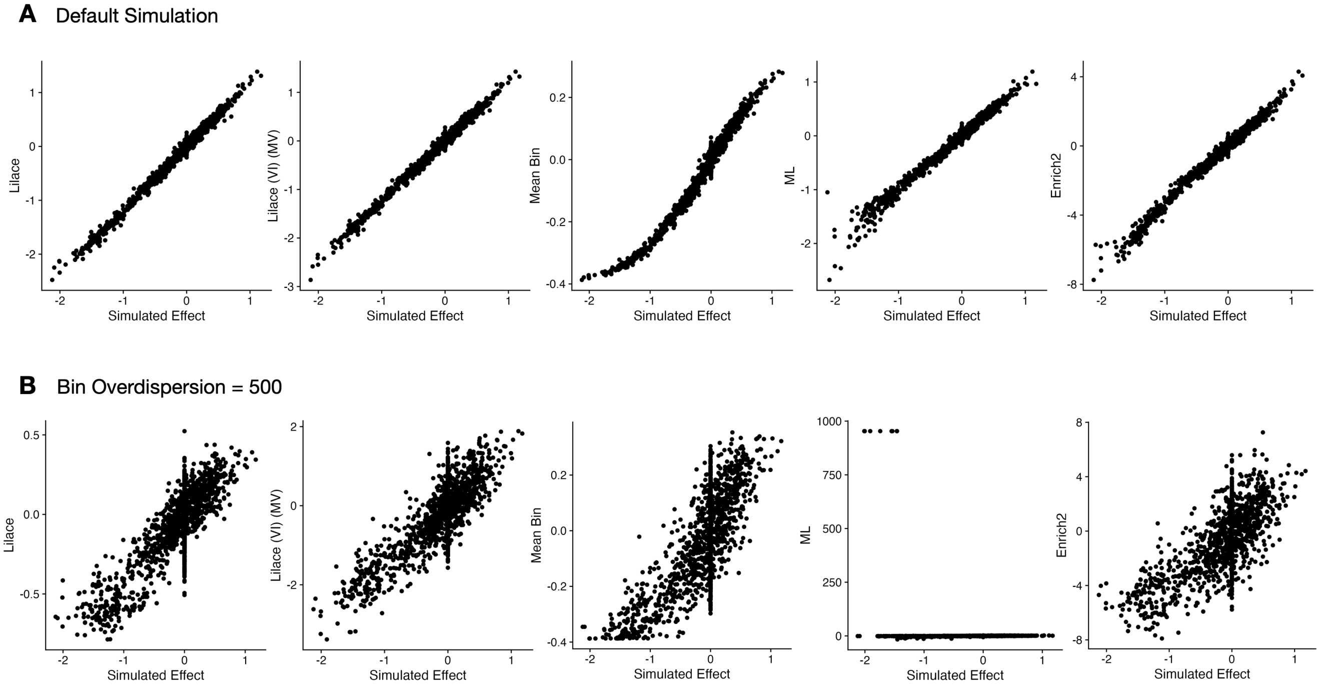

Figure S11: Scatter plots with simulated ground truth for (A) default simulation and (B) simulation with bin overdispersion = 500.

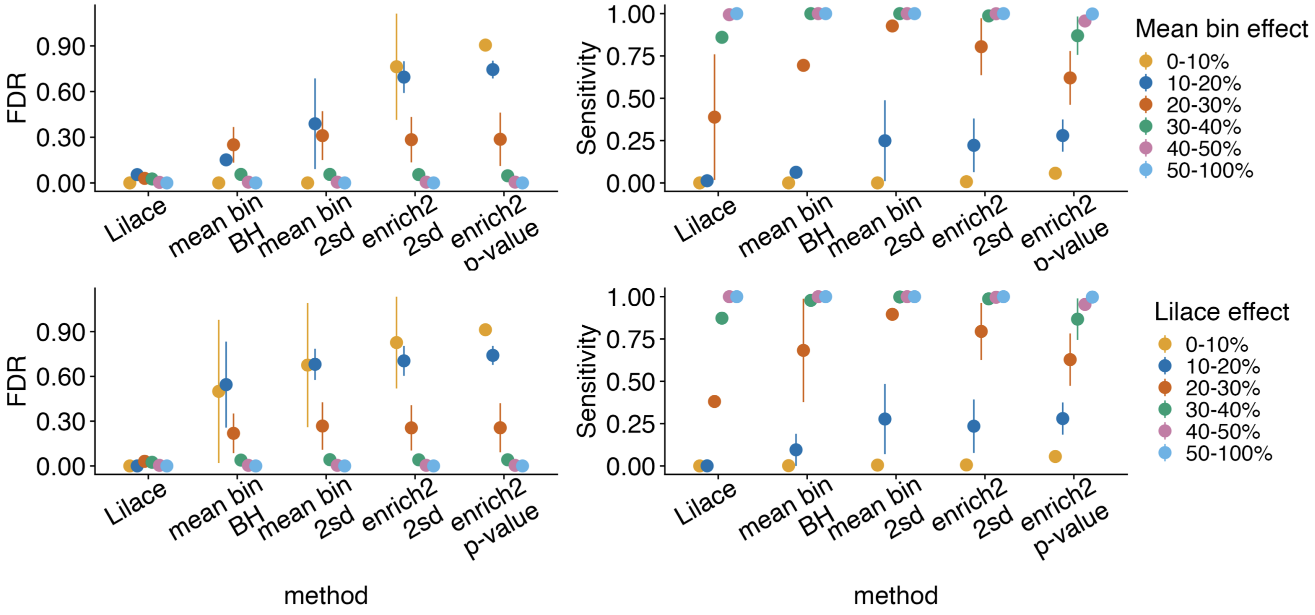

Figure S12: FDR and Sensitivity of different methods partitioned by the estimated effect size percentile (computed only on variants with a true simulated effect), using both (A) mean bin and (B) Lilace effect estimates. The inability of the mean bin BH approach to consider replicate variance leads to increased FDR near its cutoff. The approaches that do consider this (Lilace and Enrich2) do not suffer from the same issue. At lower Lilace-estimated effect sizes, all approaches other than Lilace have high FDR.

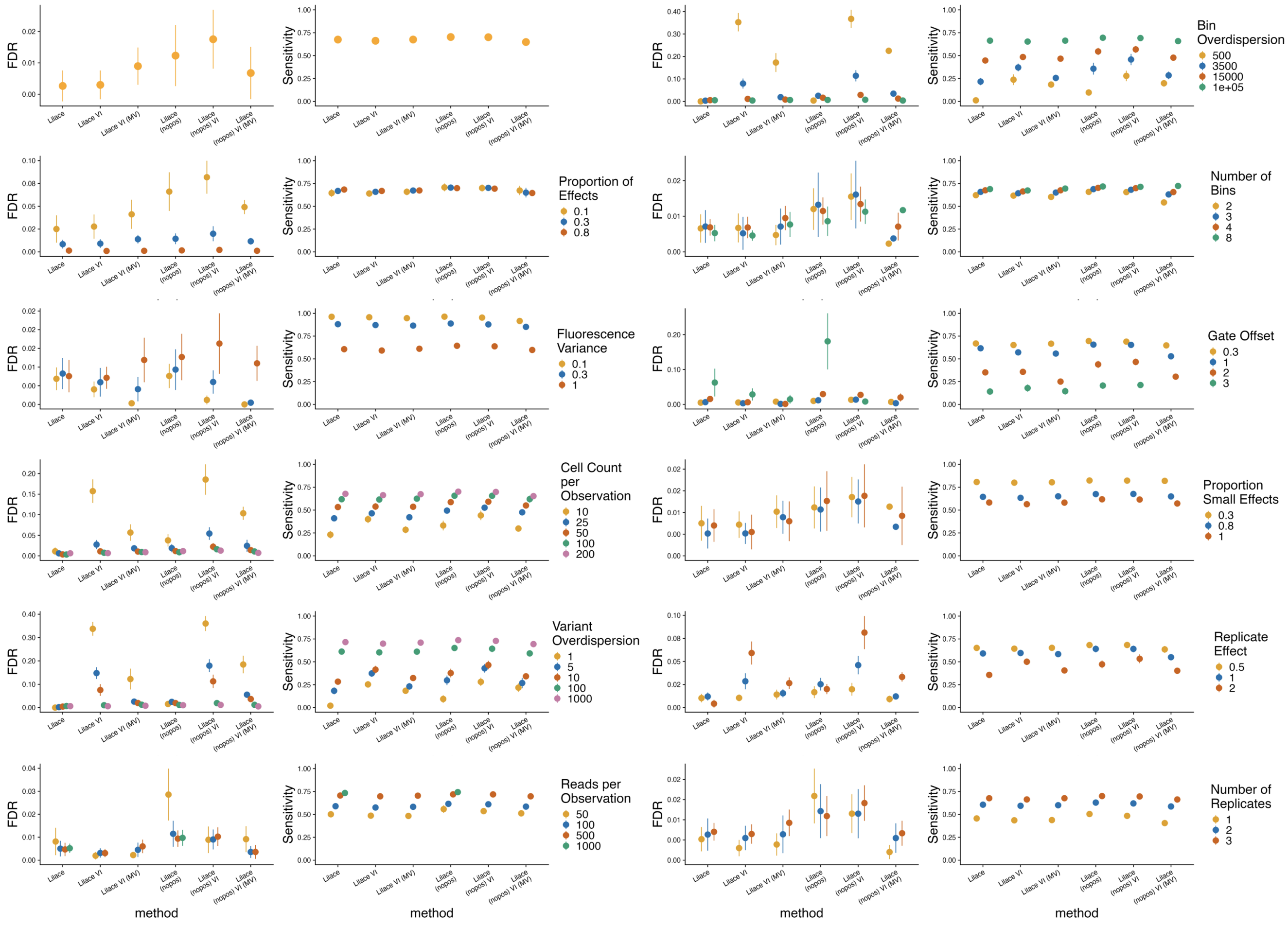

Figure S13: OCT1-based simulation FDR and Sensitivity results comparing Lilace with its variational approximations, as well as the version of the models without the position effect.

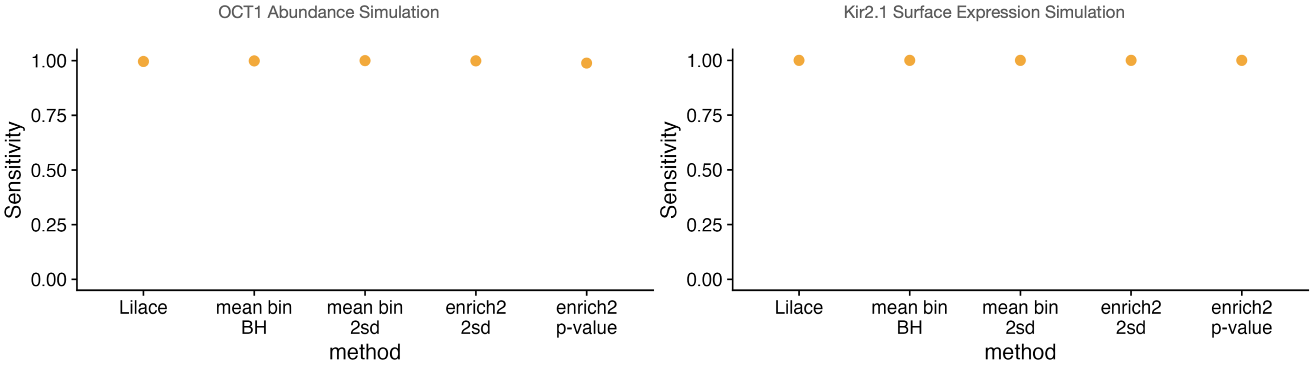

Figure S14: Default simulation sensitivity results filtered to the top 50% of effects

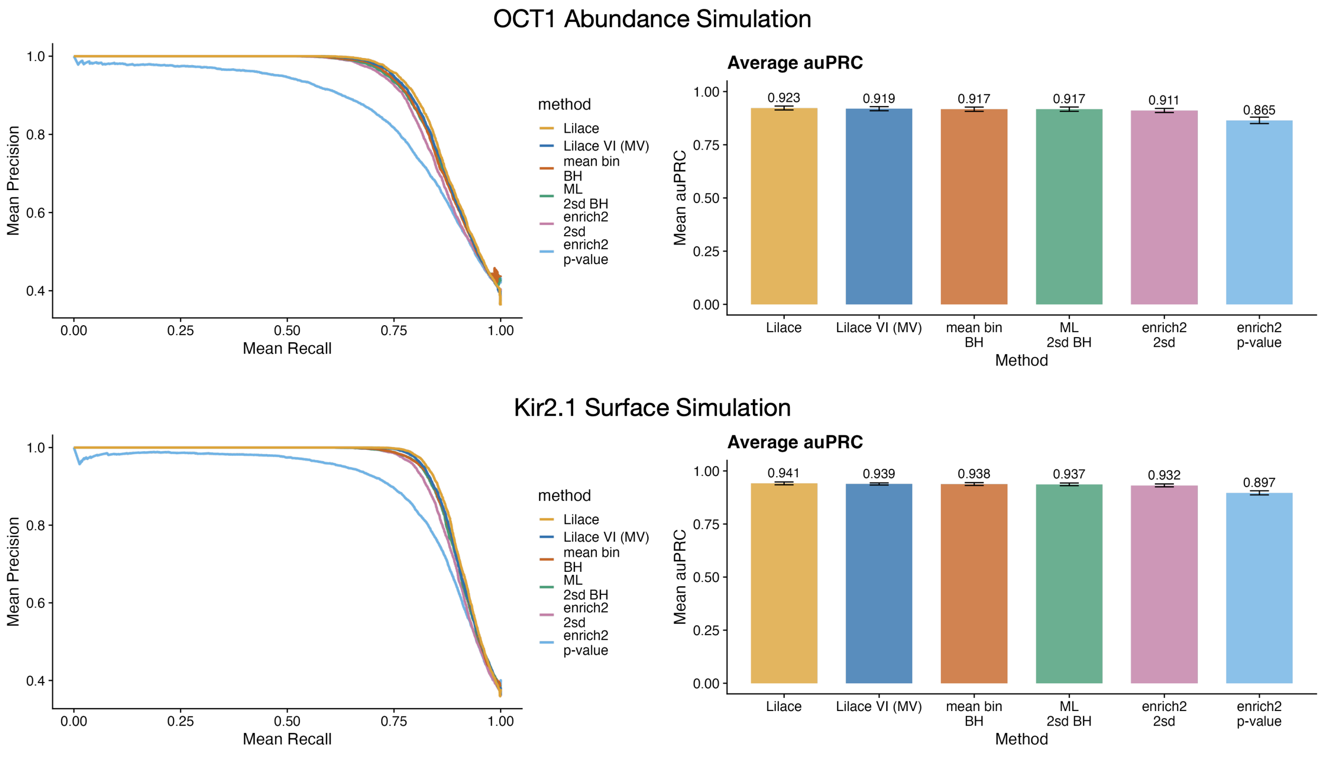

Figure S15: Average precision-recall curves (PRC) and area under the precision recall curves (auPRCs) for the default OCT and Kir2.1 simulations. Overall performance is very similar, though Lilace slightly edges out other approaches by this metric, achieving the highest sensitivity for a given FDR level, especially at the more relevant FDR values.

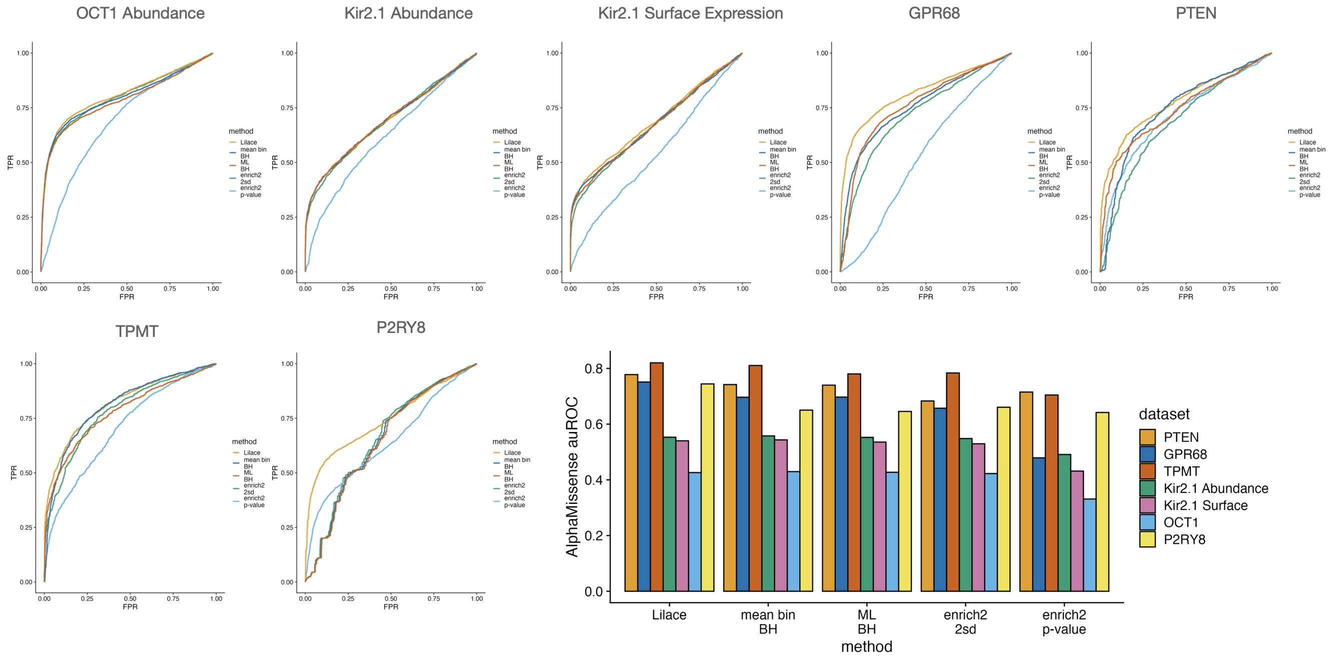

Figure S16: Receiver-Operater curves (ROC) for each approach based on AlphaMissense pathogenicity levels and associated auROCs. Lilace is the only method that is consistently a top performer in each dataset.

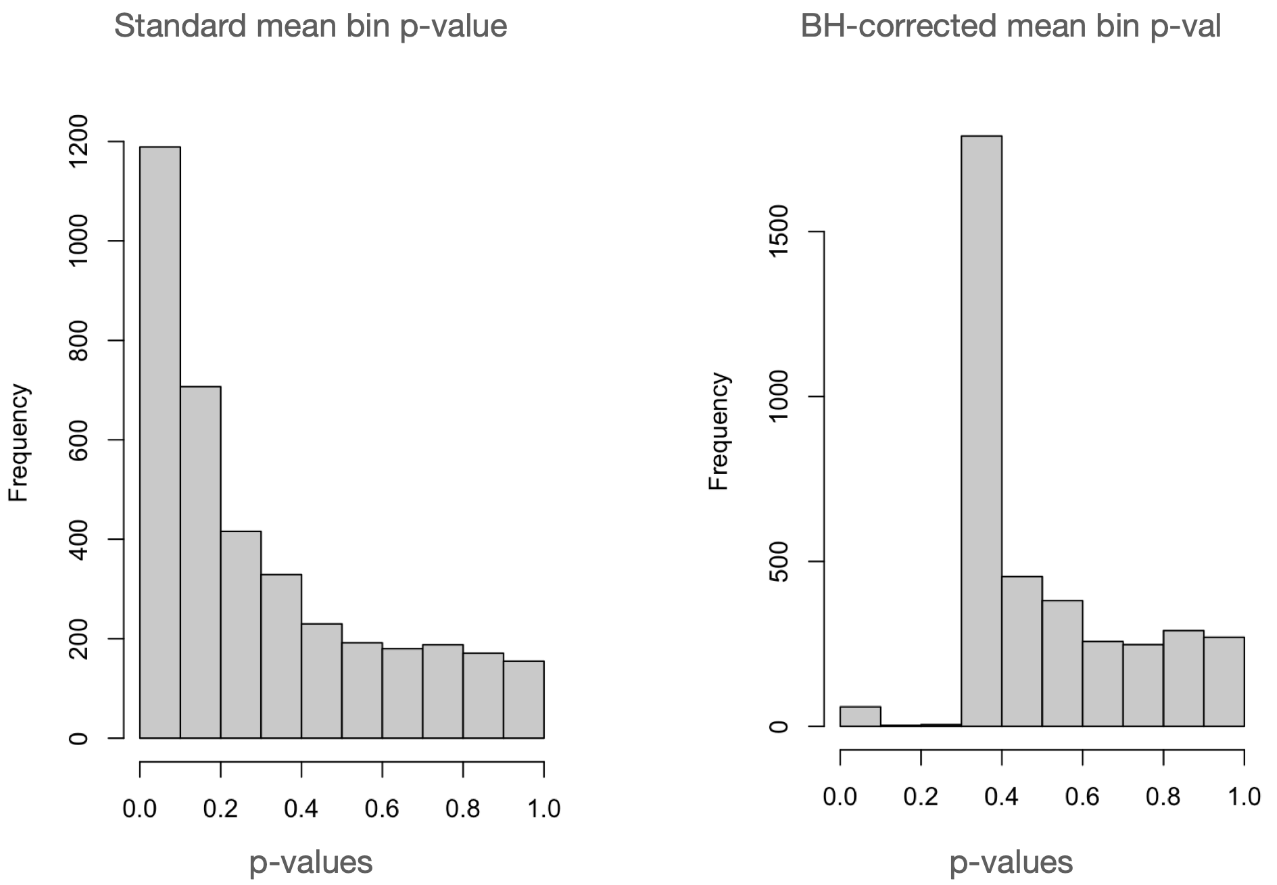

Figure S17: Comparison of raw versus BH-corrected mean bin p-values in the PTEN dataset. In this case, the BH correction vastly reduces the discovery set.

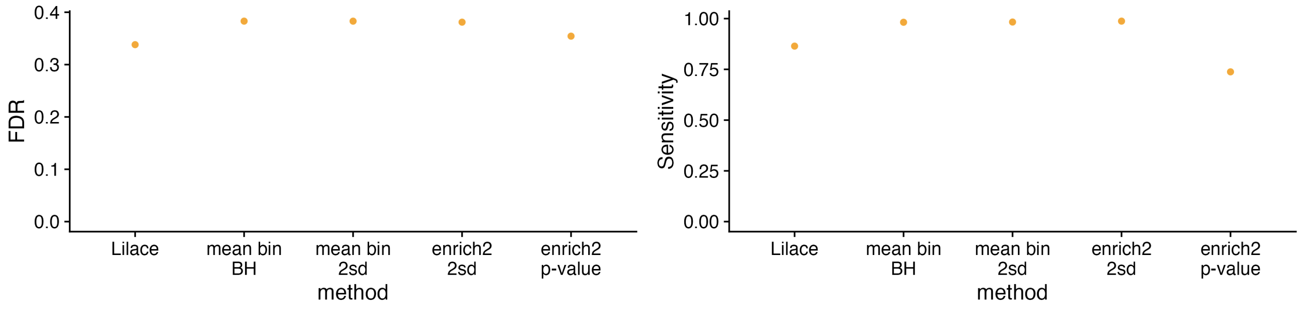

Figure S18: P2RY8 AlphaMissense-based FDR and sensitivity comparisons

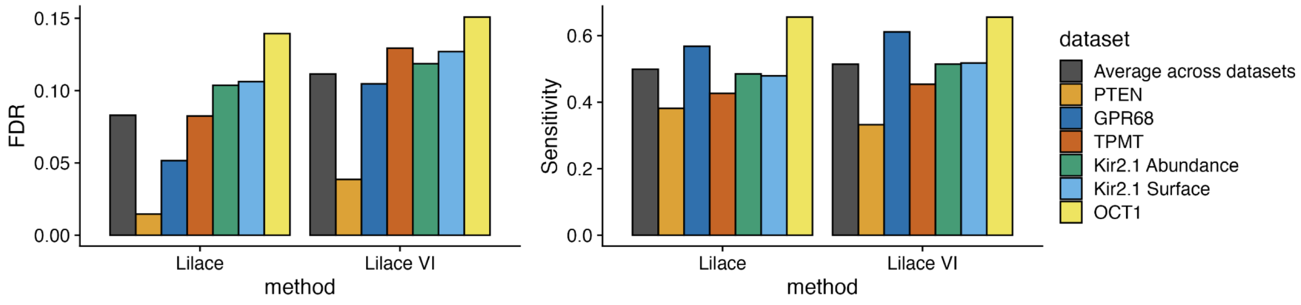

Figure S19: AlphaMissense-based FDR and sensitivity results comparing Lilace with the variational implementation of Lilace. We can see the variational version has similar sensitivity across the board but slightly higher FDR in some datasets.

**Dataset**	**Lilace Correlation**	**Mean bin correlation**	**ML correlation**	**Enrich2 correlation**
OCT1	**-0.61**	**-0.61**	-0.59	-0.59
Kir2.1 surface	**-0.38**	**-0.38**	-0.37	-0.34
Kir2.1 abundance	**-0.36**	-0.35	-0.35	-0.31
GPR68	**-0.70**	-0.63	-0.59	-0.57
PTEN	**-0.44**	-0.34	-0.40	-0.35
TPMT	**-0.62**	**-0.62**	-0.59	-0.59
P2RY8	-0.59	-0.59	-0.59	-0.59

Table S1: Scoring approach correlations with AlphaMissense pathogenicity scores (higher AlphaMissense scores indicate higher pathogenicity probability)

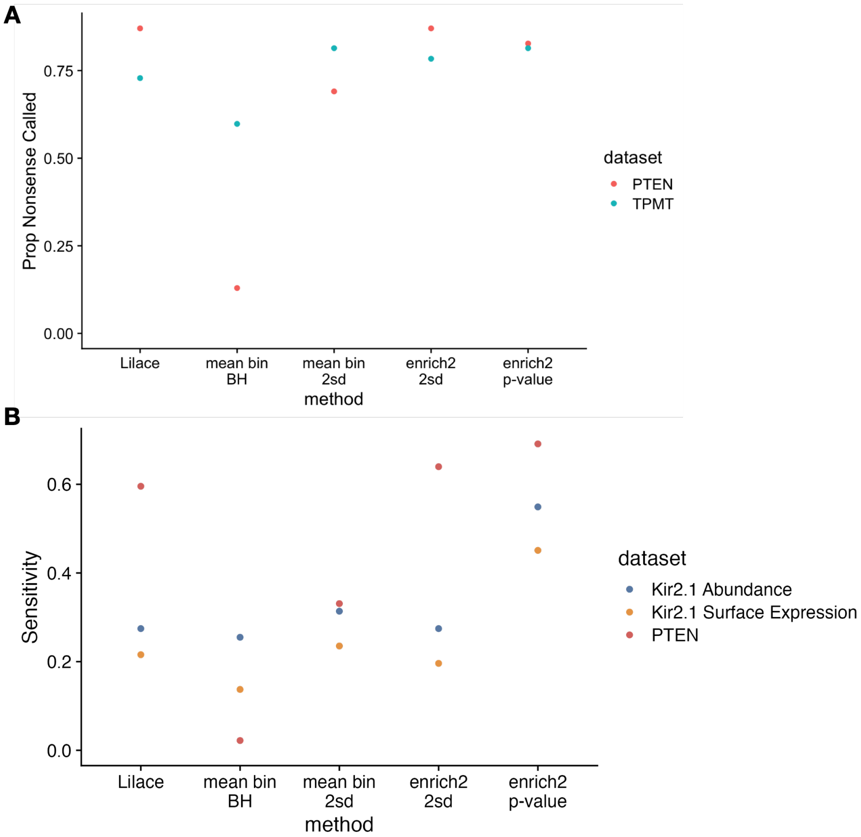

Protein	# Benign Missense	# Pathogenic Missense
OCT1	6	0
Kir2.1	2	51
GPR68	3	1
PTEN	5	224
TPMT	3	0
P2RY8	0	1

Figure S20: (A) Nonsense and (B) pathogenic ClinVar sensitivity checks when available. Only Kir2.1 and PTEN had enough labeled ClinVar variants to be used for this metric.

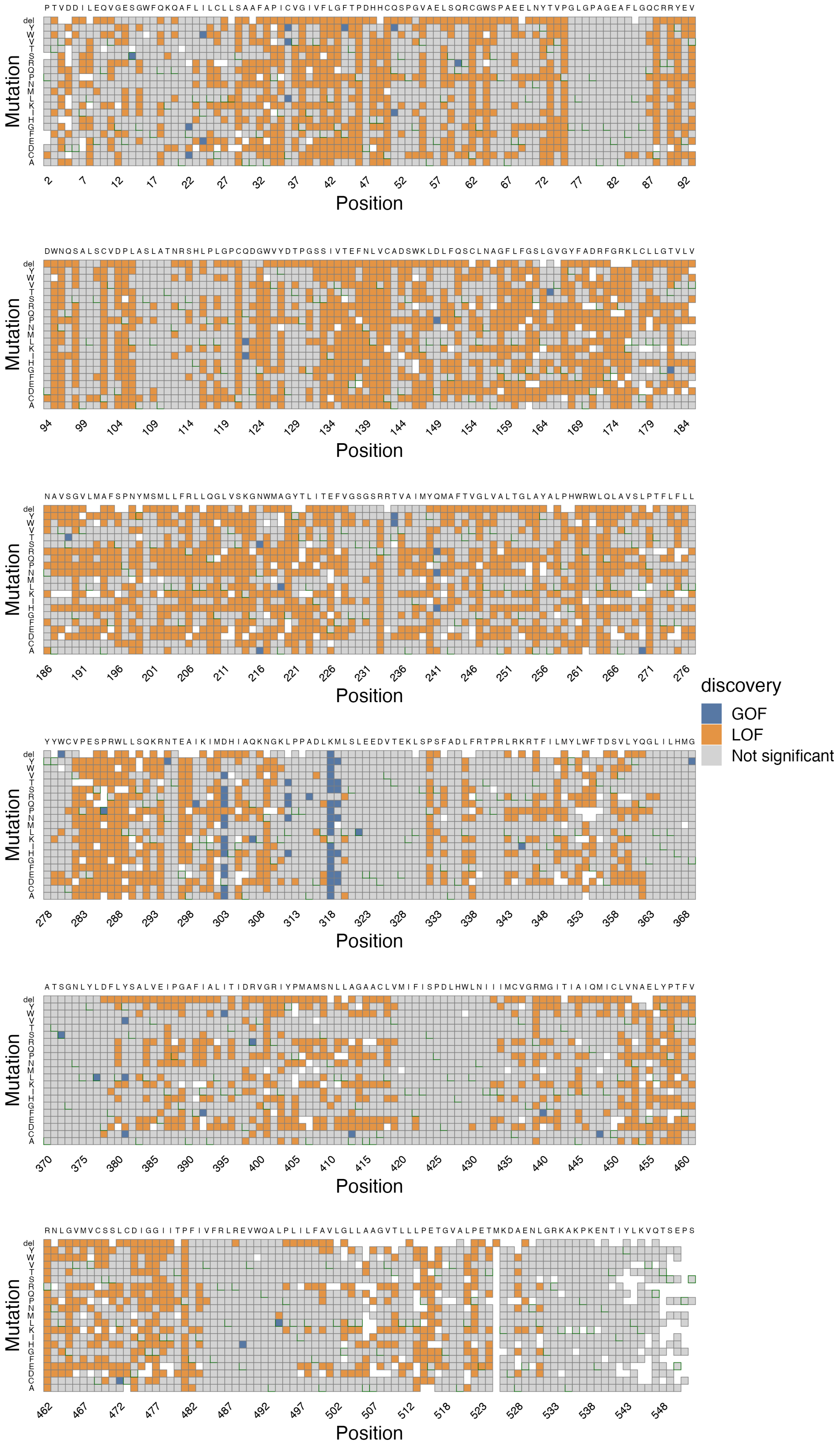

Figure S21: OCT1 full Lilace discovery heatmap

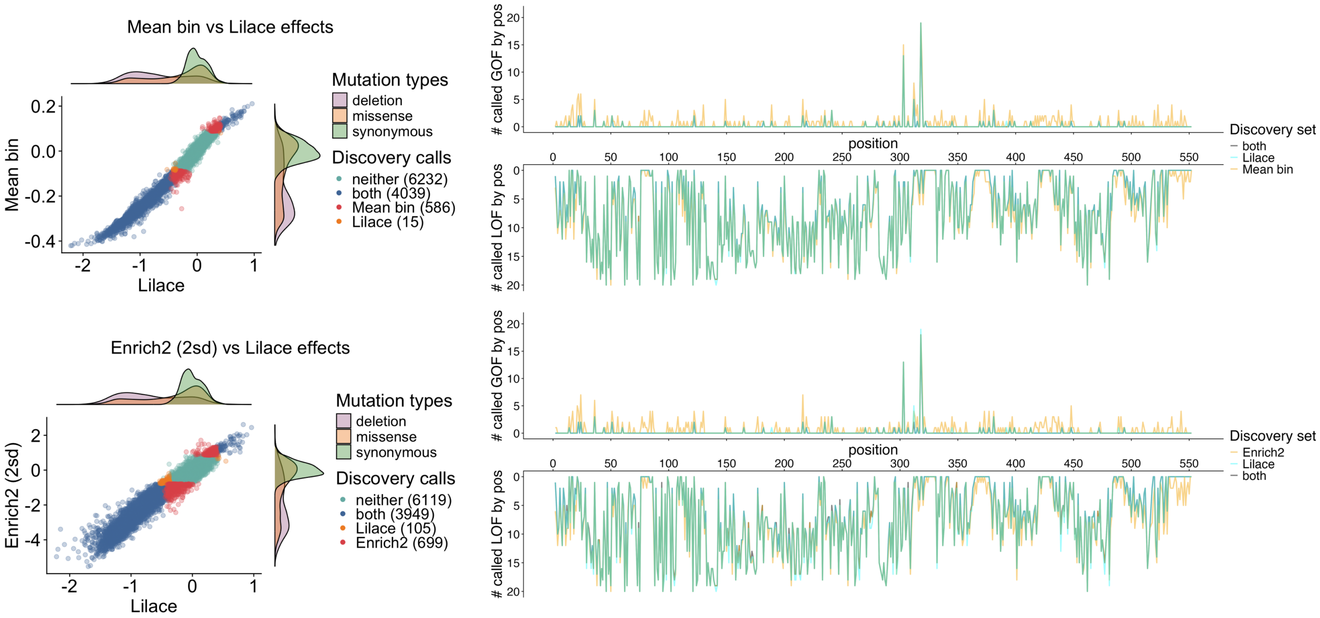

Figure S22: OCT1 mean bin and standard deviation-based Enrich2 discovery call comparisons with Lilace

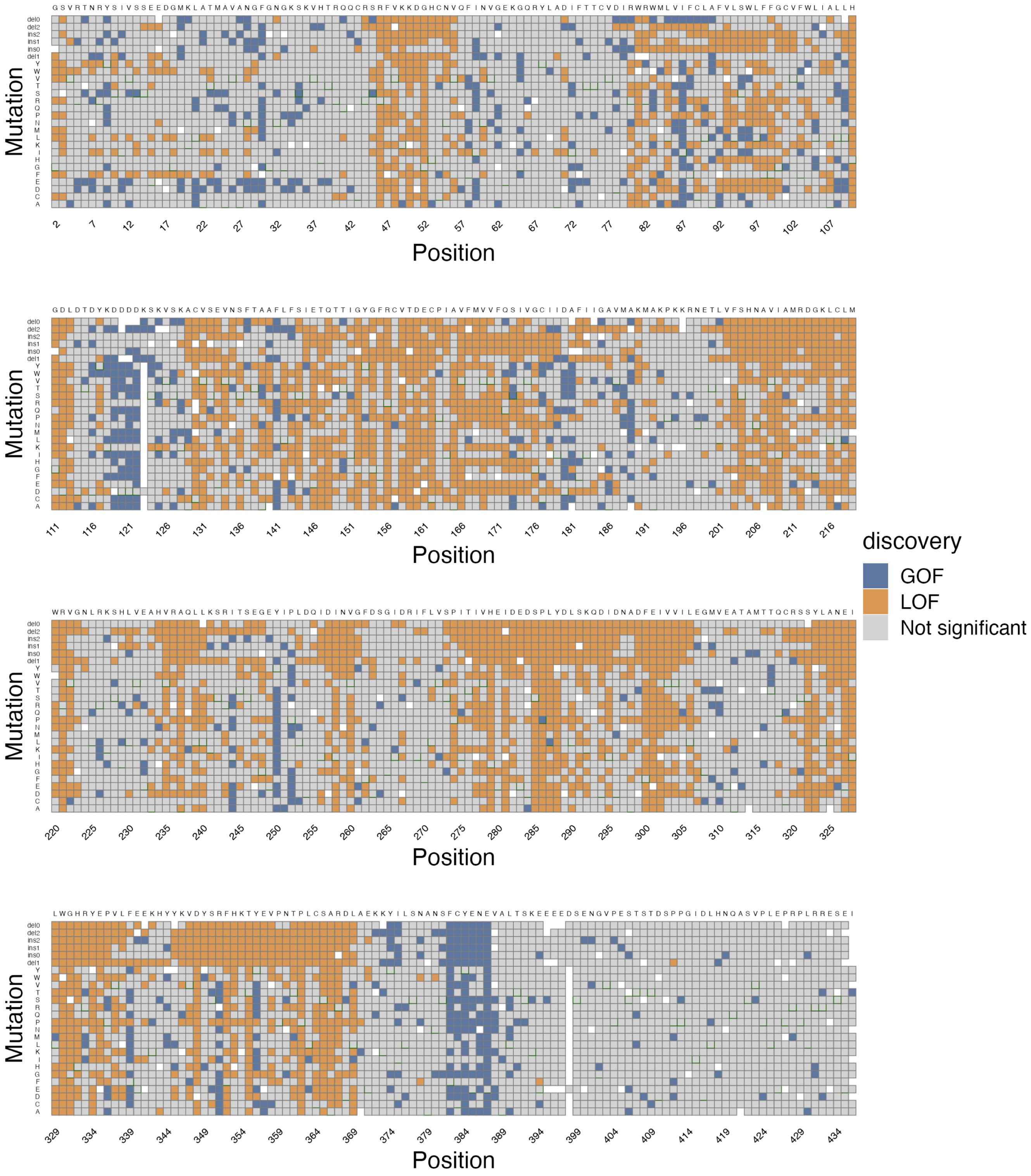

Figure S23: Kir2.1 Abundance Lilace discovery heatmap

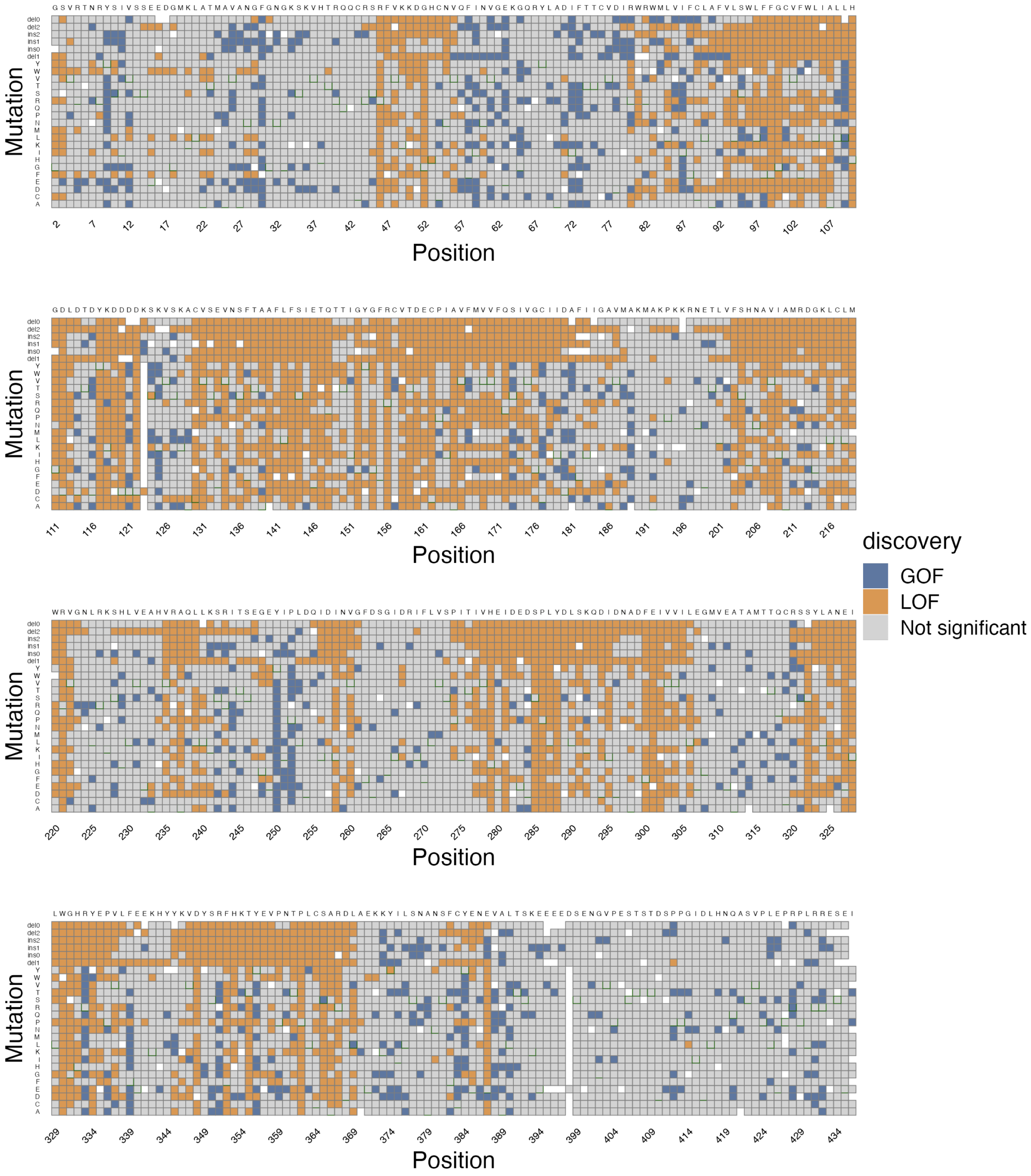

Figure S24: Kir2.1 Surface Expression Lilace discovery heatmap

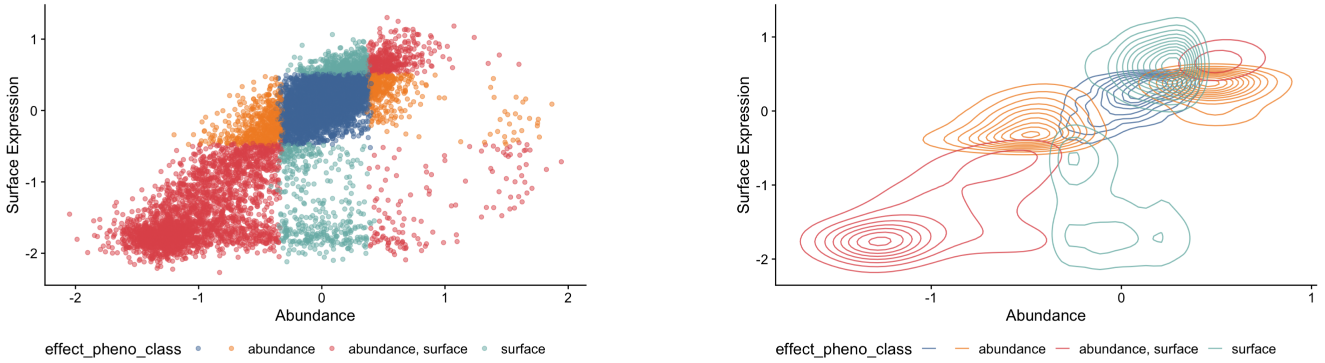

Figure S25: Kir2.1 Lilace phenotype comparison

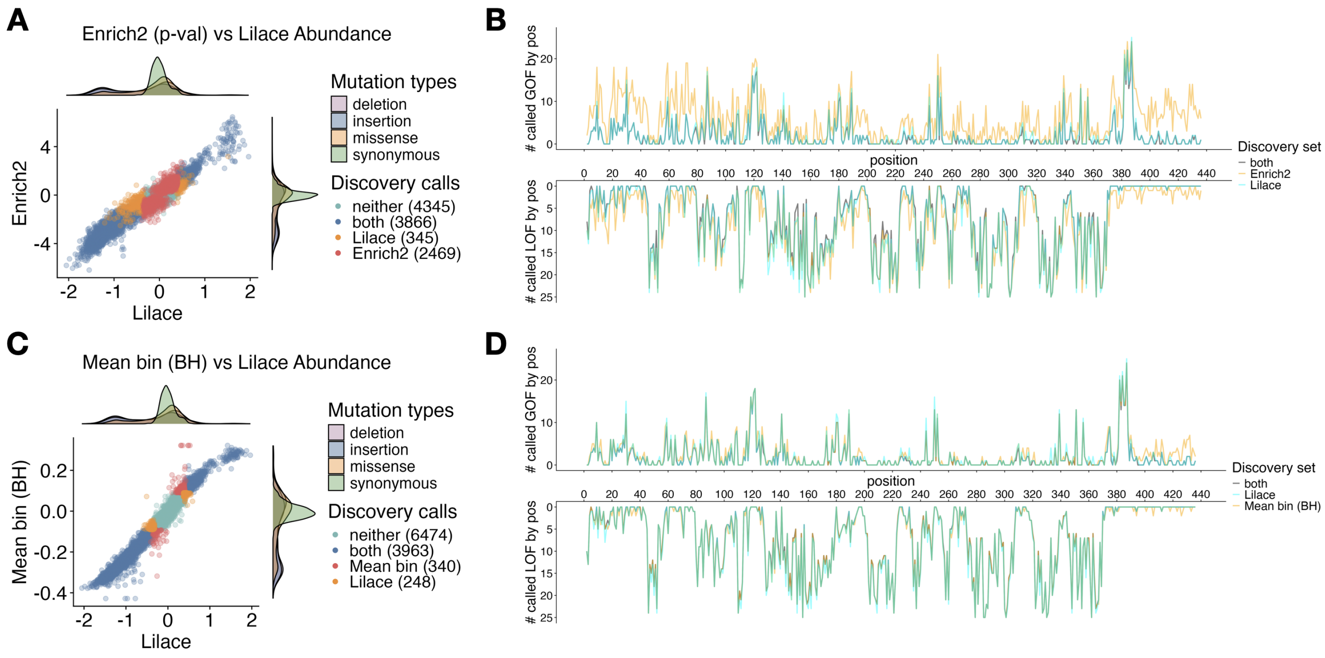

Figure S26: Kir2.1 Abundance method comparison with Lilace

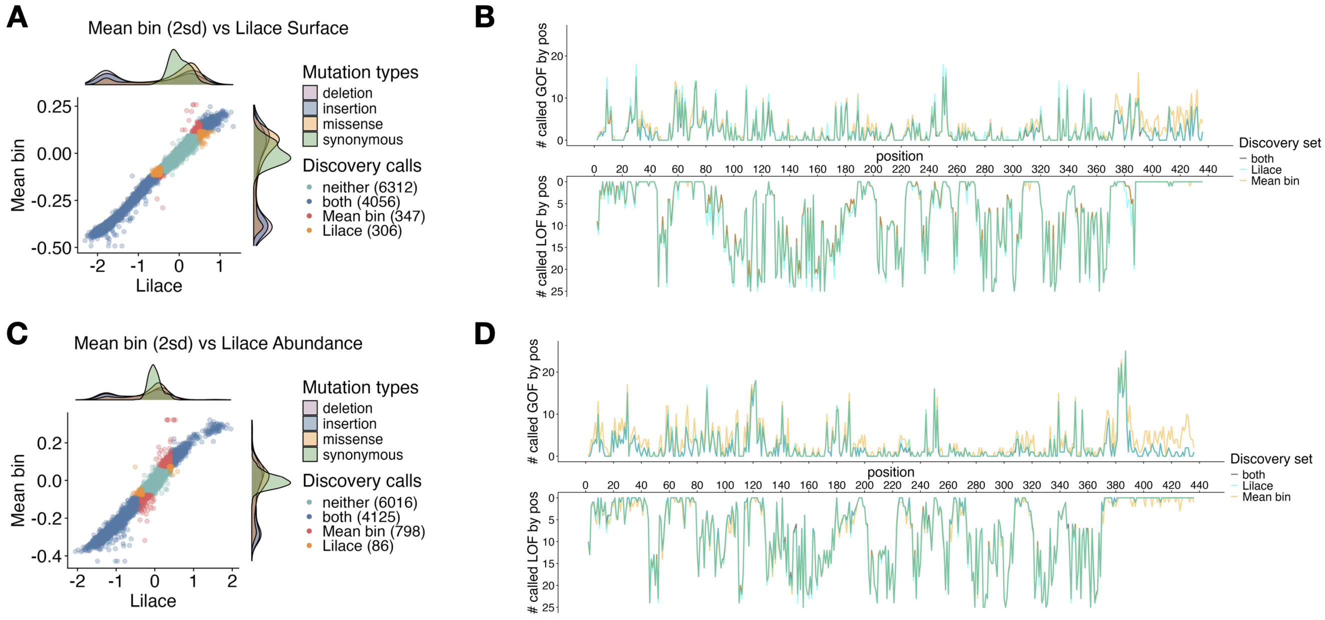

Figure S27: Kir2.1 standard mean bin (2 sd cutoff) comparison with Lilace

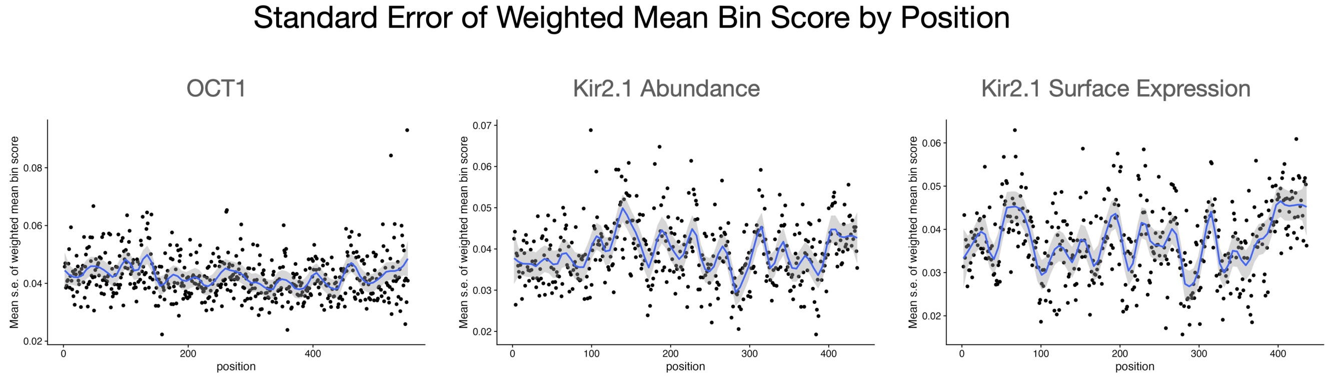

Figure S28: Average standard errors of Weighted Mean Bin Score by position. Standard error is computed as standard deviation of the score across the three replicates. Smoothed line is fit with a loess curve with a span of 0.1.

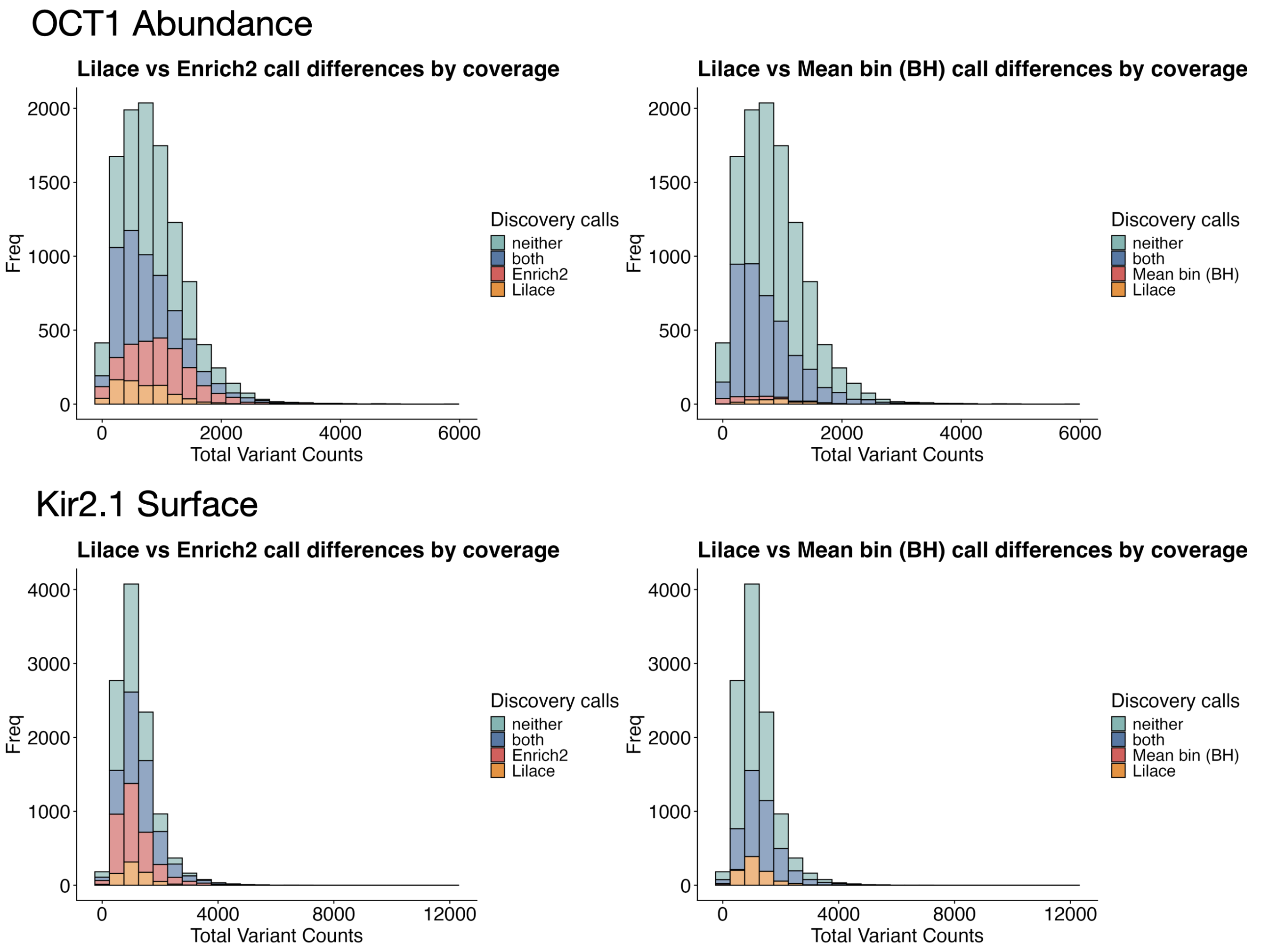

Figure S29: Stacked histograms showing read coverage distribution of different method calls.

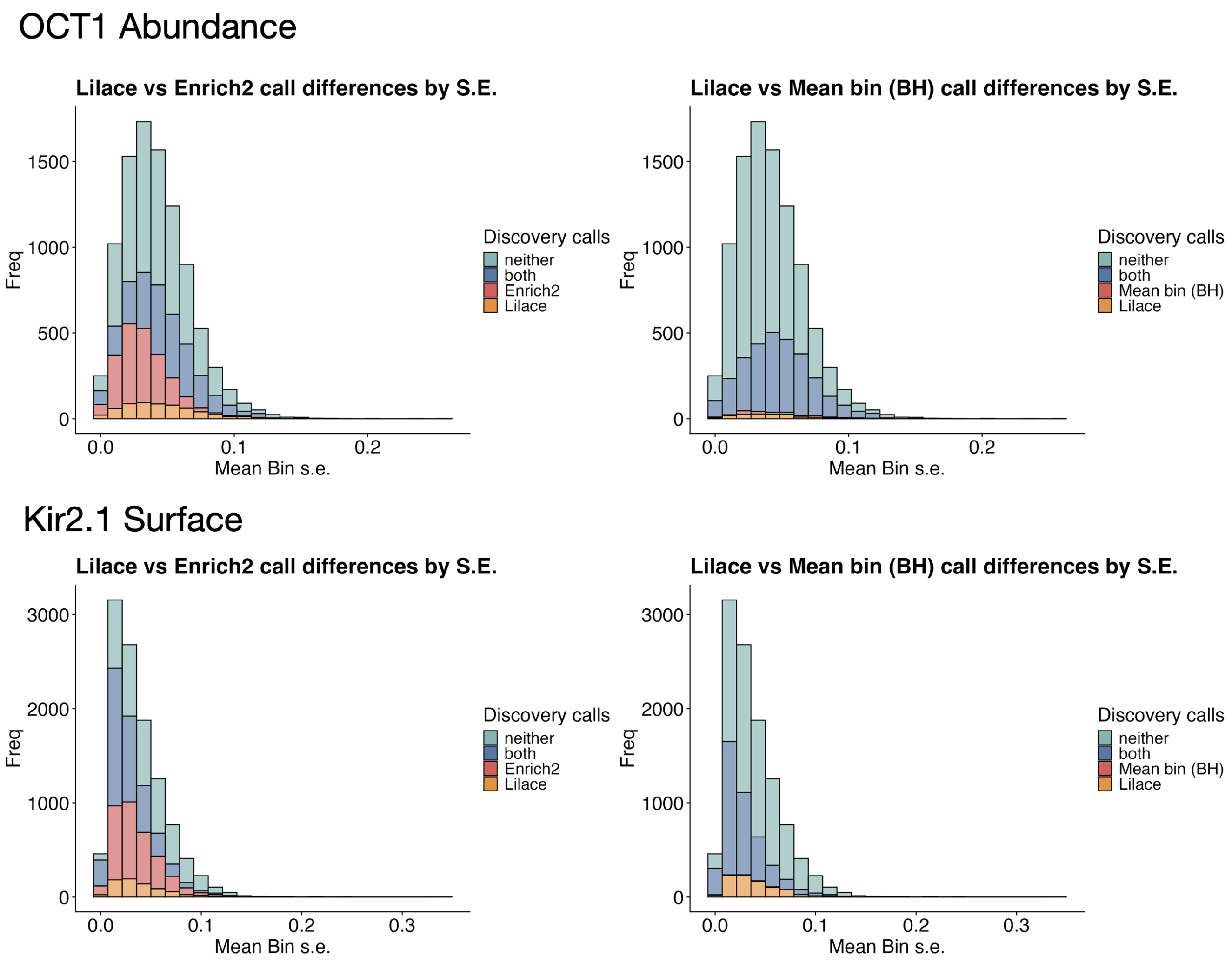

Figure S30: Stacked histograms showing mean bin standard error distribution of different method calls.

Method	Abundance Only	Surface Expression Only	Both
Lilace	901	1050	3308
Enrich2 p-value	1991	2164	4339
Mean Bin 2sd	1468	948	3448
Mean Bin BH	1383	593	2914

Table S2: Kir2.1 joint phenotype discovery numbers

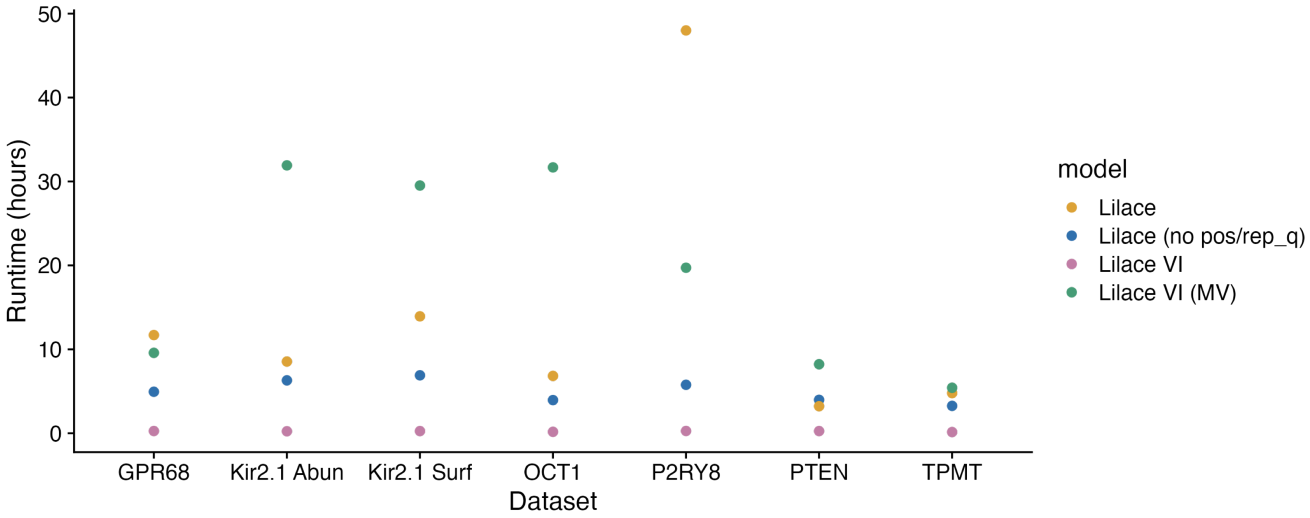

Figure S31: Runtime comparison of Lilace and its variational version on different datasets on UCLA's computing cluster. As a comparison, we also plot Lilace (no pos/rep_q), which is a version of Lilace without a position effect or replicate-specific baseline parameter. For most datasets, the additional position and replicate-specific parameters do not substantially increase runtime. On the computing cluster, the VI version was not able to fully utilize Pyro's inbuilt parallelization, so would run much faster if given more resources (on the order of minutes on our local M1 MacBook for both guide types).

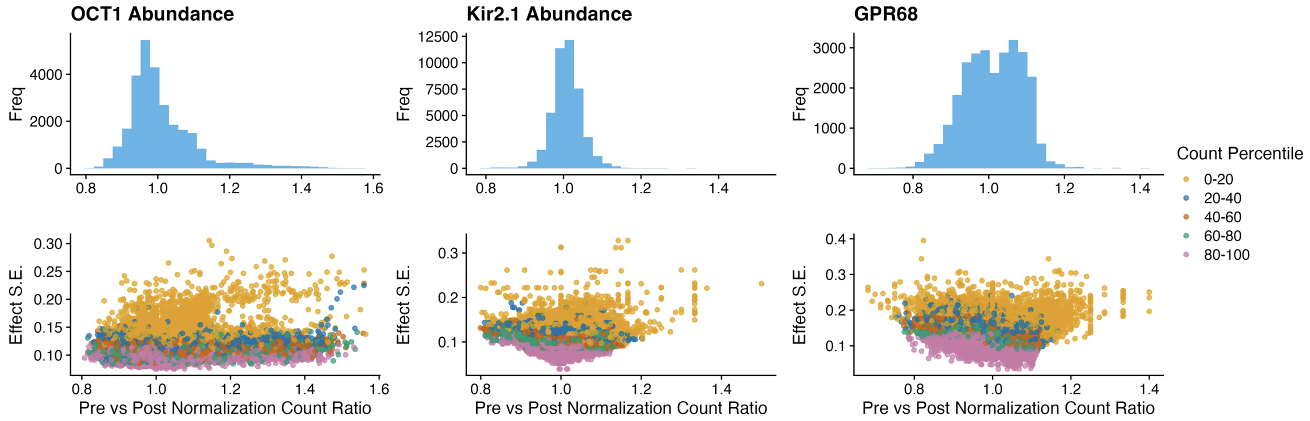

Figure S32: Effect standard errors versus the ratio of post- and pre-normalization counts for a variant observation, colored by pre-normalization count percentile. As the ratio deviates from 1, we observe larger standard errors. The normalization ratio histogram is provided for reference—the majority of variants live in the regime that is not excessively affected by the normalization.

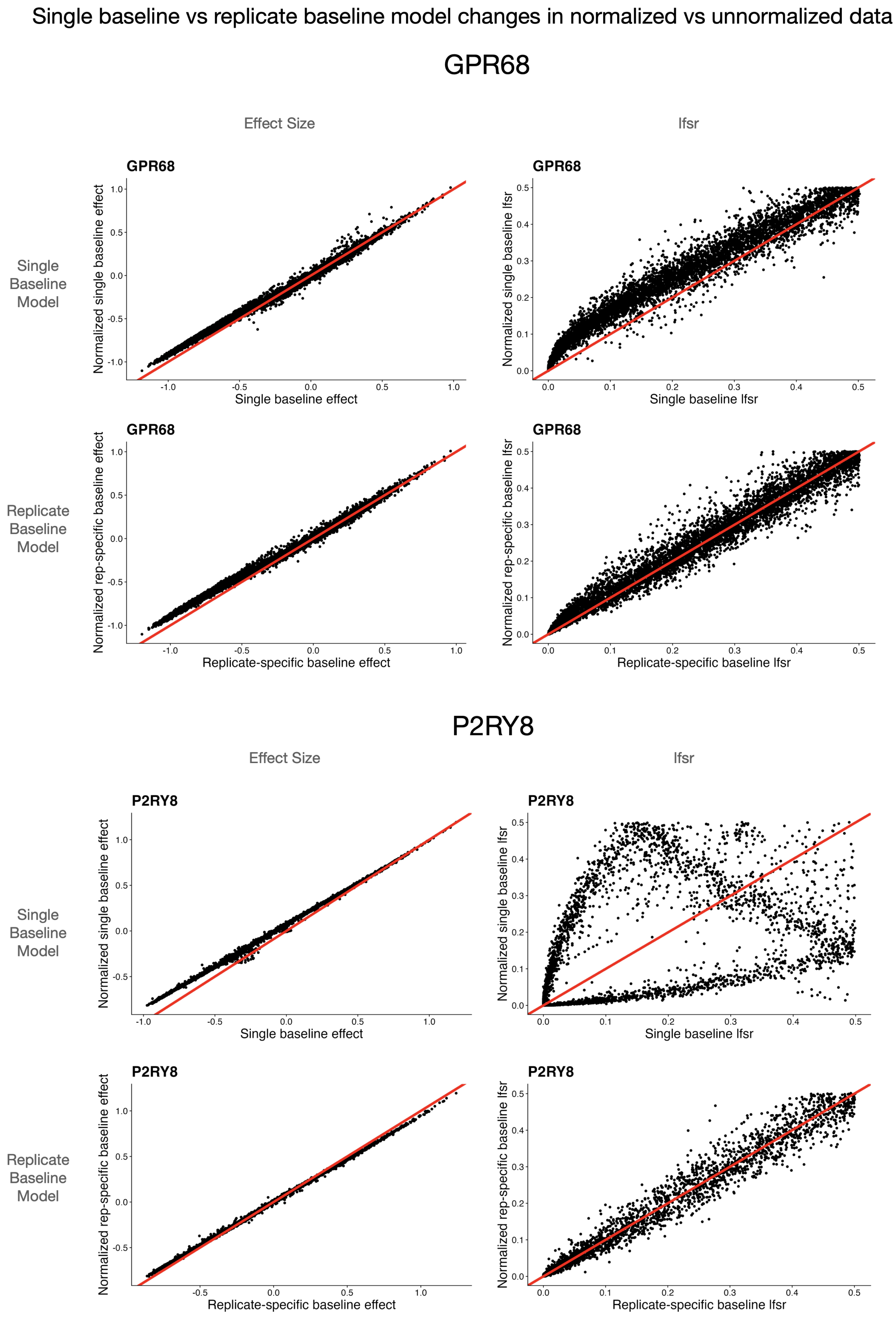

Figure S33: Side-by-side comparison of the impact of cell proportion normalization on the results of single baseline Lilace and replicate-specific baseline Lilace. The y-axis of each plot represents the model result on the normalized data and the x-axis the unnormalized data. In these datasets, using the replicate specific baseline is able to mostly recover the effect sizes and lfsrs of the normalized data without the sorting information needed for normalization. Even in the case of GPR68 where the effect sizes are still shifted by normalization, the lfsrs are corrected.
